# Toll-like receptor 3 activation enhances antitumor immune response in lung adenocarcinoma through NF-κB signaling pathway

**DOI:** 10.3389/fimmu.2025.1585747

**Published:** 2025-05-08

**Authors:** Ang Li, Man Luo, Xiyao Liu, Hongjiao Wu, Xiaoguang Liu, Zhi Zhang, Xuemei Zhang

**Affiliations:** ^1^ School of Public Health, North China University of Science and Technology, Tangshan, China; ^2^ College of Life Science, North China University of Science and Technology, Tangshan, China; ^3^ Hebei Key Laboratory of Occupational Health and Safety for Coal Industry, North China University of Science and Technology, Tangshan, China; ^4^ Affliated Tangshan Gongren Hospital, North China University of Science and Technology, Tangshan, China

**Keywords:** toll-like receptor 3, lung adenocarcinoma, PD-L1, tumor microenvironment, immunotherapy

## Abstract

**Background:**

Toll-like receptor 3 (TLR3) is a pattern recognition receptor known to play a crucial role in the immune response to cancer. However, its effect on the efficacy of immunotherapy in lung adenocarcinoma (LUAD) remains unclear. This study aims to investigate the role of TLR3 in LUAD by examining its expression levels, prognostic significance, and impact on immune signaling pathways.

**Methods:**

We analyzed the impact of TLR3 expression on the prognosis of lung adenocarcinoma patients using data from the Cancer Genome Atlas (TCGA) database and four additional cohorts (GSE72094, GSE30219, GSE50081 and GSE31210). Functional enrichment analyses were performed to compare molecular features between low and high TLR3 expression groups using gene set variation analysis (GSVA). We also examined the correlation between TLR3 and tumor mutation burden (TMB), immune infiltration, and PD-L1 expression. Further experimental validation was conducted using co-culture systems of LUAD cells and peripheral blood mononuclear cells (PBMCs) with PD1 inhibitors, and Western blot analysis to investigate the involvement of NF-κB signaling.

**Results:**

TLR3 expression was significantly lower in LUAD tissues compared to normal tissues, with high TLR3 expression correlating with better survival outcomes across multiple cohorts. High TLR3 expression was associated with increased TMB and enhanced immune activation. Patients with high TLR3 expression exhibited higher immune checkpoint expression and immune cell infiltration. Experimental results showed that TLR3 agonists increased the susceptibility of LUAD cells to activated PBMCs under PD1 inhibitor therapy, inhibiting cell proliferation, migration, and invasion. Additionally, TLR3 has a strong positive correlation with MHC molecules and upregulated PD-L1 expression. NF-κB was identified as a key regulator of PD-L1 expression, with TLR3 agonists enhancing NF-κB and PD-L1 activity.

**Conclusion:**

TLR3 enhances the anti-tumor immune response in LUAD by modulating NF-κB signaling and PD-L1 expression, making it a promising prognostic biomarker and therapeutic target. This study highlights the potential of TLR3 to improve immunotherapy outcomes, providing a comprehensive analysis of its role in LUAD and paving the way for novel therapeutic strategies targeting TLR3-mediated pathways.

## 1 Introduction

Based on the 2022 Global Cancer Statistics Report, lung cancer has the highest rates of both incidence and mortality compared to all other cancer types worldwide (1). Despite advancements in early detection and treatment methods, the overall survival (OS) rate for most LUAD patients has not significantly improved (2). Recently, PD1/PD-L1 targeted immunotherapy has shown significant clinical efficacy in the treatment of non-small cell lung cancer (NSCLC) (3–6). However, studies have shown that some tumor patients have response rates of less than 40% to anti-PD-L1/PD1 monotherapy due to primary or secondary resistance to immunotherapy (7, 8). Hence, the identification of targets that can increase the sensitivity of immune checkpoint inhibitors (ICIs) therapies by activating the immune response within the tumor microenvironment is of paramount importance.

Toll-like receptors (TLRs) are a highly conserved group of type I transmembrane proteins that play a crucial role in innate immunity (9, 10). Studies have showed that TLR3 is under-expressed in various cancer tissues, and its activation can convert cancer cells from immune tolerance to anti-tumor immunity, enhancing their anti-tumor activity (11–13). Similar to other TLRs, TLR3 agonist poly(I:C) has been demonstrated to activate natural killer (NK) cells and dendritic cells (DCs) via NF-κB signaling, leading to robust antitumor responses (14). It can also enhance the recruitment and activity of immunosuppressive cell types, such as myeloid-derived suppressor cells (MDSCs), through the internal signaling pathways of tumor cells, thereby promoting the formation of an immunosuppressive environment (15, 16).

Numerous research efforts indicate that individuals with high PD-L1 exhibit significantly improved survival rates after anti-PD1 antibody treatment (17–20). However, tumor cells can enhance PD-L1 transcription by activating transcriptional regulatory factors that modulate the JAK/STAT1/IRF1, NF-κB, and JAK/STAT3 signaling pathways in response to various cytokine stimuli (21, 22). As a novel form of immunotherapy, TLR3 agonists induce a significant upregulation of PD-L1 in cells by activating CD4+ and CD8+ T cells, as well as PD-L1 blockers (23). This phenomenon has been reported in a limited number of cancer types, including neuroblastoma. Therefore, this study investigates the role of TLR3 agonists in modulating the expression of PD-L1, an immune-related molecule present on the surface of lung adenocarcinoma cells, with the aim of enhancing the efficacy of sindelimab.

Given the critical role of TLRs in immune regulation and its potential impact on PD-L1 expression and NF-κB signaling, this study aims to provide valuable insights into TLR3 as an immunotherapy target, offering new avenues for enhancing the efficacy of immunotherapies in LUAD patients.

## 2 Materials and methods

### 2.1 Cell culture

A549 and PC9 cells were purchased from Procell (Hubei, China). These cells were cultivated in RPMI-1640 medium supplemented with 10% fetal bovine serum and antibiotics. The culturing environment was a humidified incubator set at 37°C and 5% CO_2_.

### 2.2 Chemicals and reagents

Sintilimab is an anti-PD-1 antibody supplied by Innovent Biologics (Jiangsu, China). TLR3 agonist Poly(I:C) was obtained from Invitrogen (CA, USA). NF-κB inhibitor BAY 11-7082 was purchased from MedChemExpress (NJ, USA). T Cell activator for activation of PBMCs were bought from StemCell Technologies(VAN, CAN). For cell culture, the fetal bovine serum (FBS) was obtained from Tianhang Biotechnology (Zhejiang, CHN). For western blot analysis, the antibody against β-actin was purchased from Proteintech (Chicago, IL,USA). The human NF-κB p65, NF-κB p65 (phospho S536), IKB alpha, IKB alpha (phospho S36), TLR3 and PD-L1 were purchased from Abcam(Cambridge, UK).

### 2.3 Migration and invasion assay

To conduct cell migration assays, 200 μL of FBS-free medium containing cells (3 × 10^4^ cells) was placed into a 24-well Transwell culture insert. In the case of cell invasion assays, 1 × 10^5^ cells were introduced into the upper chamber that was lined with 0.5% Matrigel (Corning Incorporated, Corning, USA). Subsequently, a total of 600μL of the complete medium was introduced into the lower chamber. Cells that moved to the bottom of the chamber or invaded it were preserved with 4% paraformaldehyde for 30 minutes and then dyed with 0.5% crystal violet (Solarbio, Beijing, China) for 15 minutes. At least five random fields of view were imaged under the microscope and cells was recorded.

### 2.4 Cell proliferation assays

For cell proliferation assays, cells from different treatment groups (5×10^3/^well) were cultured in a 96-well plate and allowed to incubate for 24, 48, and 72 hours. The viability of the cells was assessed using the Cell Counting Kit-8 (Mei5 Biotechnology, Beijing, CHN). In the colony formation assay, cells from different treatment groups (3×10^5/^well) were distributed into a six-well plate and cultured for 2 weeks in medium with 10% FBS. Colonies were subsequently treated with 4% paraformaldehyde for 20 minutes and then stained using 0.1% crystal violet (Solarbio, Beijing, China) for 15 minutes. Following this, the colonies were captured in photographs and subjected to analysis.

### 2.5 Quantitative real-time PCR

RNA from the cells was isolated with TRIZOL reagent (Invitrogen, CA, USA) and subsequently reverse-transcribed into cDNA using the RevertAid first-strand cDNA synthesis kit (Thermo Fisher Scientific, MA, USA). After this step, use SYBRGreen PCR Master Mix (Vazyme, China) for real-time quantitative PCR. The reverse transcription and qRT-PCR reaction setups were arranged following the guidelines provided by the manufacturer. To determine the relative levels of gene expression, the 2^(−ΔΔCT) method was employed, with normalization to *GAPDH* expression levels for every sample. The primers can be found in **Supplementary Table 1**.

### 2.6 Western blot and primary antibodies

Cells were disrupted using RIPA buffer (Thermo Fisher Scientific, MA, USA), and the total protein concentration was determined employing a BCA kit (Solarbio Science and Technology, Beijing, CHN). 10% SDS-PAGE was utilized to separate proteins, which were then transferred onto nitrocellulose membranes. The membranes underwent blocking with 5% milk at room temperature for a duration of 2 hours. After the blocking step, the membranes were exposed to the specified primary antibody, followed by a 1h incubation at 37˚C with a secondary antibody conjugated to horseradish peroxidase (HRP). Imaging was carried out using enhanced chemiluminescence luminescence reagents (Seven Biotech, Beijing, CHN). *β-actin* served as the reference control. All antibodies, including anti-*NF-κB p65*, anti-*NF-κB p65* (phospho S536), anti-*IKB* alpha (phospho S36), Anti-*TLR3*, and Anti-*PD-L1*, were obtained from Abcam (Eugene, USA).

### 2.7 Construction of PD-L1 promoter plasmid and dual-luciferase reporter assay

We created reporter constructs that contained a 1424 bp fragment from the promoter region of *PD-L1*. The primers used were *PD-L1*-PF (5′-GG GGTACC TTT ATG CCC TGG GTC TTG-3′) and *PD-L1*-PR (5′-CCG CTCGAG TGA CCT TCG GTG AAA TCG-3′), these constructs featured cloning sites for *Kpn I* and *Xho I* (NEB, Ipswich, USA), as indicated by the underlined sequences. Subsequently, the PCR product was inserted into the pGL3-basic reporter vector (Promega, Madison, USA). Verification of all constructs was performed through direct sequencing. LUAD cells (2 × 10^5^)were seeded in 24-well plates. Upon reaching 80% confluence, the cells were transfected using 200ng of pGL3-PD-L1_pro_ and 1ng of pRL-SV40 plasmid with Lipofectamine 2000 reagent (Thermo Fisher Scientific, USA). Subsequently, the cells were then exposed to either 20 mM of the *NF-κB* inhibitor or a control for an additional 24 hours. After the treatment period, cells were harvested and the fluorescence signal was measured using a dual-luciferase reporter assay (Promega, USA).

### 2.8 Electrophoretic mobility shift assay

Isolation of nuclear proteins from A549 cells through the use of NE-PER™ Nuclear and Cytoplasmic Extraction Reagents (Thermo Fisher Scientific, Waltham, USA). The capacity for DNA binding was evaluated with a LightShiftTM Chemiluminescent EMSA kit (Thermo Fisher Scientific, Waltham, USA). The probes corresponding to the putative binding site of *NF-κB* on the promoter of *PD-L1* were synthesized by Sangon Biotech (Shanghai, China). The sequences were *PD-L1*-PF (5′-AGC TTT CAA AAG GGC TTT CTT AAC CCT CAC C-3′) and *PD-L1*-PR (5′-GGT GAG GGT TAA GAA AGC CCT TTT GAA AGC T-3′). For regular EMSA, the binding reaction components were added in order according to the manufacturer’s instructions. During this phase, 20μg of nuclear extracts were treated with 20fmol of biotin-labeled probes for a duration of 15 minutes. To verify the specificity of the interactions between DNA and proteins, unlabeled competitive probes (cold probe and mutant probe) were employed. Additionally, the binding ability was verified using A549 nuclear protein treated with *NF-κB* inhibitor (BAY 11-7082). The reactions involving binding were carried out using electrophoresis in a 6.5% polyacrylamide gel. Following this, the samples were placed onto a nylon membrane with a positive charge and were cross-linked using UV light at a wavelength of 254 nm during the cross-linking process. Finally, chemiluminescence was used to visualize.

### 2.9 Tumor cells and PBMCs co-culture system

Peripheral blood samples were diluted with an equal volume EasySepTM Buffer and slowly added to SepMate tubes containing Lymphoprep™ (StemCell Technologies, VAN, CAN). The samples underwent centrifugation at 2000 rpm for a duration of 30 minutes at room temperature, which ranged from 15 to 25°C, with the brake disabled. PBMCs were cultivated in RPMI-1640 medium supplemented with 10% fetal bovine serum and stimulated using the T Cell activator for a duration of 3 days. Activated PBMC were incubated with LUAD cells at a 5:1 effector to target ratio for 24 hours. The PBMCs/A549 cocultured cells were treated with either anti-*PD1* antibody (50µg/ml sintilimab) or Poly(I:C).

### 2.10 Bioinformatic analysis

The mutation annotation format (MAF) was obtained from the TCGA database through the utilization of the “maftools” R package, allowing us to examine the mutational landscape of patients categorized into different TLR3 expression groups.

Cox regression analysis was performed using the “survival” package to assess the association of variables with OS. The “survminer” package was employed to create Kaplan-Meier curves and perform log-rank tests.

A set of 50 hallmark gene sets (h.all.v7.5.symbols.gmt) and Kyoto Encyclopedia of Genes and Genomes (KEGG) pathways (c2.cp.kegg.v7.2.symbols) retrieved from the MsigDB database was estimated using gene set variation analysis (GSVA). Significant pathways between different groups were visualized using “ComplexHeatmap” R package.

To estimate cellular abundance in the TME, we used microenvironmental gene profiles associated with specific immune cell subsets from the previous study. The enrichment of 24 types of immune cells within the TME was assessed using GSVA (24). The immune checkpoint distribution was analyzed, and determine immune/stromal scores of tumor tissues using the “ESTIMATE” R package (25). We also analyzed the correlation between the expression of immunomodulatory factors including chemokines, immunoinhibitor, immune stimulators, histocompatibility complex (MHC), and TLR3 mRNA level in LUAD.

The “pRRophetic” R package was employed to predict the chemotherapeutic sensitivity for each lung adenocarcinoma sample using default parameters (26, 27). For immunotherapy, we used published data set of anti-PD1 and anti-CTLA4 cohorts (GSE91061) with melanoma (28), anti-PD-L1 cohort of urothelial carcinoma (IMvigor210). Subclass mapping for predicting clinical response to ICIs (29).

### 2.11 Statistical analyses

Statistical evaluations and graphical representations were carried out utilizing GraphPad Prism 8.0 (GraphPad Software, CA, USA) and R version 3.6.1. Correlation analyses were conducted using Pearson correlation analysis. Student’s t-test was used for two-group comparisons, with results presented as mean ± SD. The estimation of overall survival was performed using the Kaplan–Meier technique in conjunction with Cox regression analysis. *P* < 0.05 is the threshold for statistically significant differences.

## 3 Results

### 3.1 TLR3 expression and its clinical significance in LUAD

**Figure 1:**
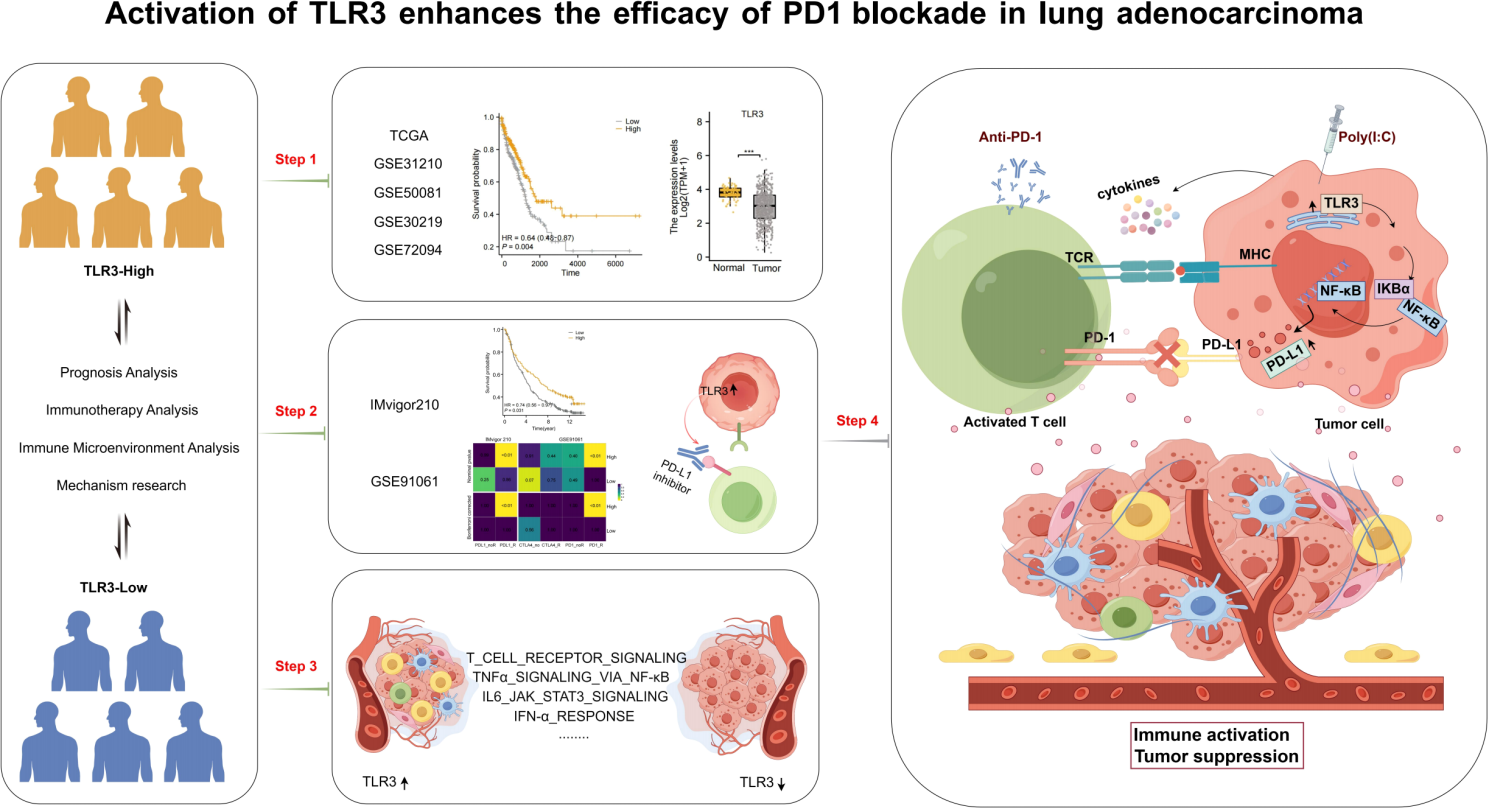
Workflow of our study. ***P<0.001.

**Figure 2:**
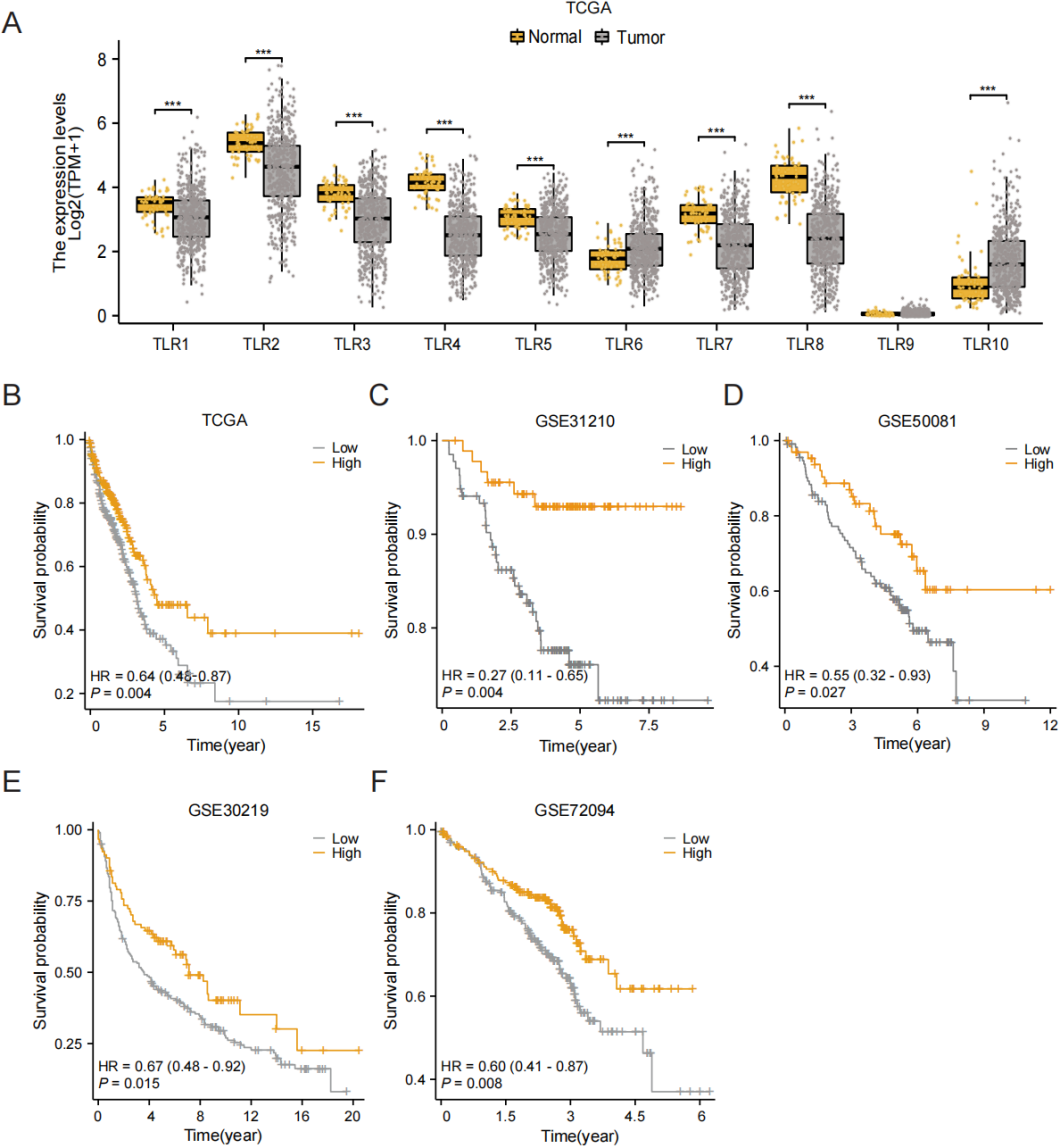
High TLR3 expression is associated with good prognosis in LUAD. **(A)** TLR3 expression in normal tissue and lung adenocarcinoma using TCGA dataset. **(B-F)** Kaplan-Meier plots for TLR3 expression based on datasets from TCGA, GSE31210, GSE50081, GSE30219 and GSE72094. ***P < 0.001.

The workflow of this research is shown in **Figure 1**. Based on data from TCGA, our analysis revealed that the expression levels of TLR3 in LUAD tissues were notably reduced compared to those in normal tissues (**Figure 2A**). Subsequent studies on the clinical outcomes of TLR3 and LUAD across six cohorts (TCGA, GSE72094, GSE13213, GSE14814, GSE11969, and GSE68465) showed that high TLR3 expression is associated with better survival (**Figures 2B–F**).

**Figure 3:**
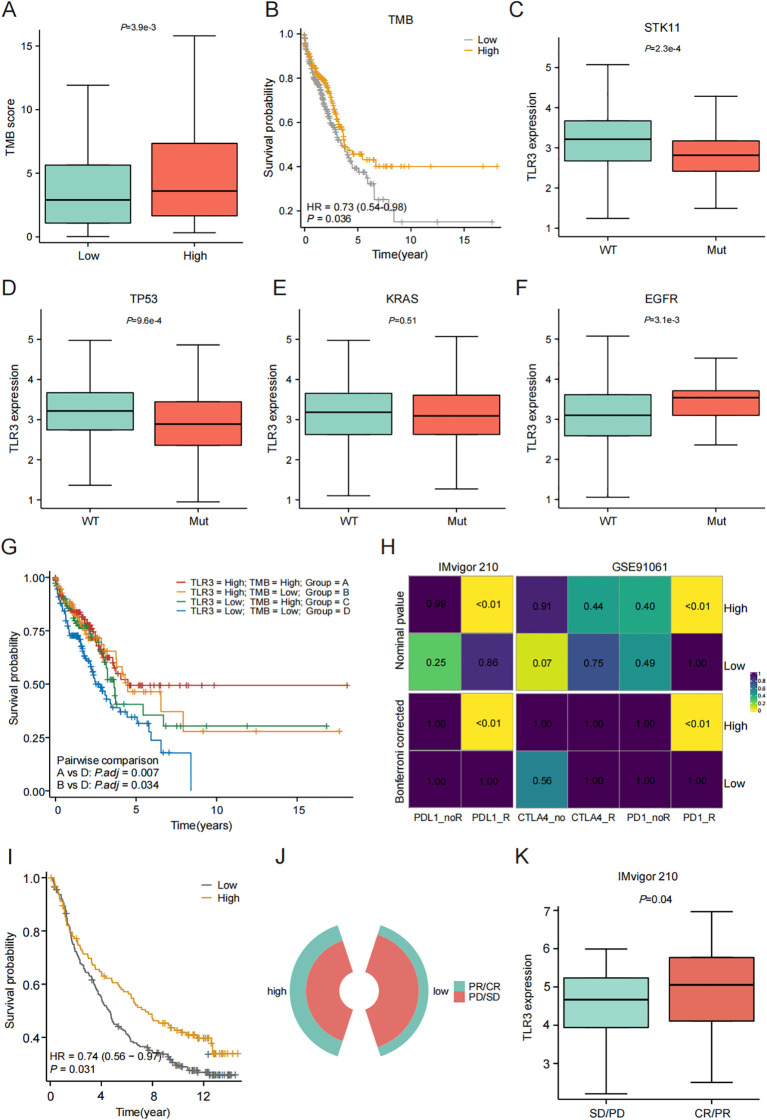
Evaluation of TLR3 expression on immunotherapy response. **(A)** Comparison of TMB between high- and low-TLR3 expression groups. **(B)** Kaplan-Meier curve of OS for patients classified by TMB. **(C-F)** The TLR3 expression in relation to mutation of STK11, TP53, KRAS and EGFR. **(G)** TMB combined with TLR3 expression to predict the prognosis of LUAD patients. **(H)** The subclass mapping algorithm predicts response to immunotherapy between high- and low-TLR3 expression groups. **(I)** Survival analyses for low and high TLR3 expression groups in Imvigor210 cohort. **(J, K)** Comparison of TLR3 expression between SD/PD and CR/PR groups of in IMvigor210 cohort. CR, complete response; PD, progressive disease; PR, partial response; SD, stable disease. Data shown as mean ± SD.

Variations in immune infiltration and expression patterns of immune-related genes across LUAD clusters suggest that the response to immunotherapy may differ among these groups. TMB is predictive biomarkers of therapeutic response to immune checkpoint inhibitors (ICIs). In our investigation of the relationship between TMB and TLR3, we observed that levels of TMB were markedly elevated in the group with high TLR3 expression (*P* < 0.05) (**Figure 3A**). Patients with higher TMB scores had better survival outcomes compared to those with lower TMB scores (**Figure 3B**). Additionally, the influences of TP53, KRAS, EGFR and STK11 mutations on TLR3 were also investigated using GSE72094 dataset. LUAD patients with wild type of TP53 and STK11 had higher TLR3 expression when compared to those with mutations, while the EGFR mutation showed the opposite trend (**Figures 3C–F**). To explore the synergistic or antagonistic potential of TMB and TLR3 in predicting survival, patients were categorized based on these factors. Patients with both high TMB and TLR3 had the most favorable prognoses, while those with low TMB and TLR3 had the worst outcomes (**Figure 3G**).

Furthermore, we analyzed the cohort of patients receiving immunotherapy using the subclass mapping algorithm to assess patient response to immunotherapy. The results showed that patients within the high TLR3 expression group were more likely to respond to anti-PD1 and PD-L1 therapy (**Figure 3H**). Additionally, we predicted the treatment response of TLR3 expression to immune checkpoint blockade using the anti-PD-L1 cohort of urothelial carcinoma (IMvigor210). High TLR3 expression was found to be a prognostic protective factor for patients receiving immunotherapy (**Figure 3I**). Also, TLR3 expression was higher in responders compared to non-responders (**Figures 3J–K**), indicating that patients with high TLR3 expression have increased immune sensitivity to PD-L1 blockade.

### 3.2 Molecular characteristics of high and low TLR3 expression in LUAD

Currently, in the majority of LUAD instances, classification occurs according to levels of molecular expression, potentially linking them to distinct biological functions. The hallmark gene sets, which encompass eight categories of processes, effectively summarize most of the relevant information derived from the original sets. Therefore, we explored the different molecular features of low and high TLR3 expression group.

**Figure 4:**
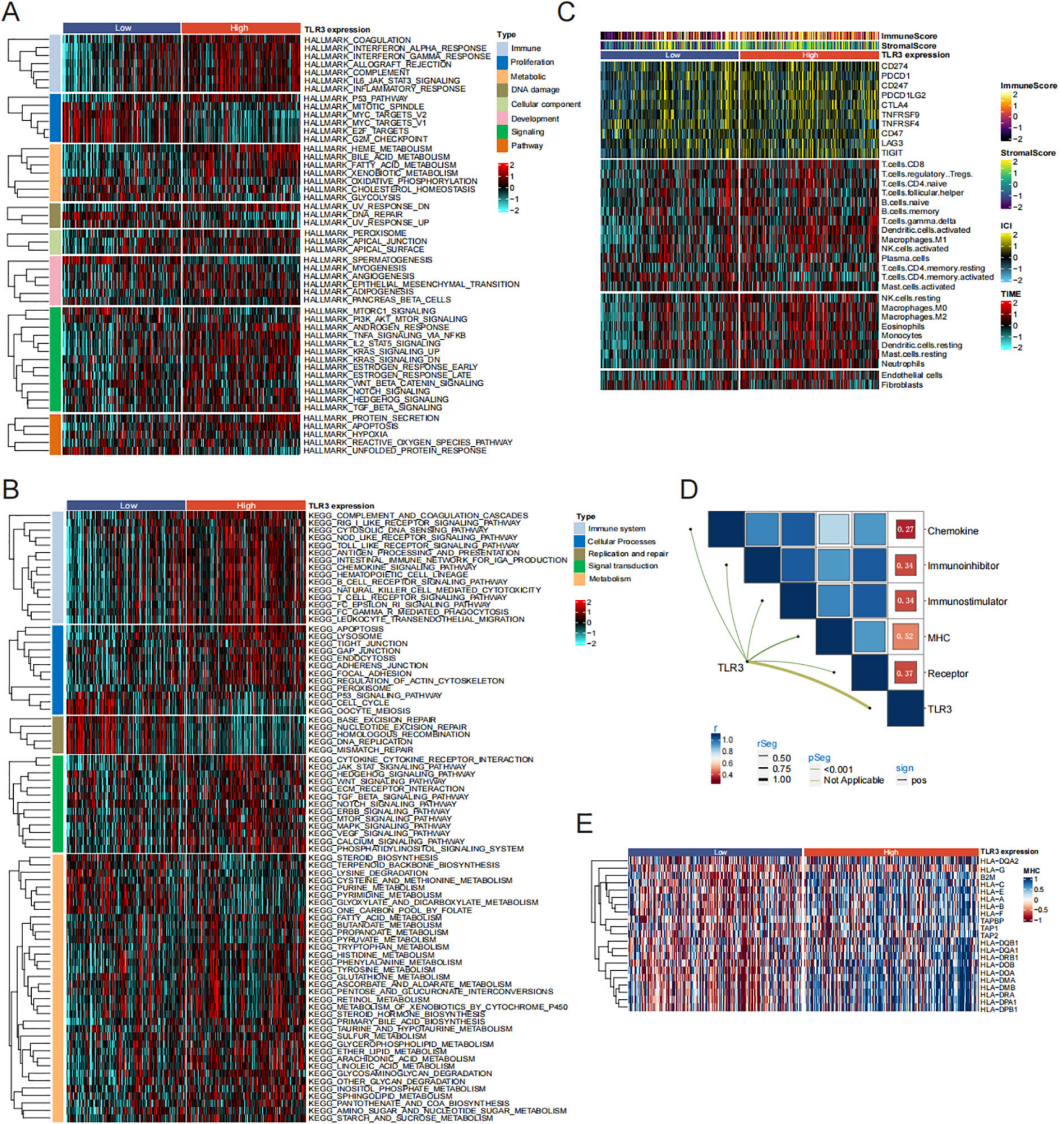
TLR3-related Pathway and immune infiltration analysis in LUAD. **(A, B)** Signal pathway enrichment analysis of 50 hallmark gene sets and KEGG analysis. **(C)** Immune checkpoint expression and immune cell infiltration enrichment in the TCGA cohort. **(D, E)** Correlation analysis between TLR3 expression and chemokine, receptor, MHC, immunoinhibitor and immunostimulator.

It was observed that low-TLR3 expression exhibited the most activation in proliferation-related pathways (G2M checkpoint, E2F targets and Mitotic spindle), whereas those with high TLR3 expression had the lowest activation in these pathways. Conversely, patients with high TLR3 expression demonstrated the most immune activation (Complement, interferon alpha response, interferon gamma response and IL6-JAK-STAT3 signaling) and signaling-related processes (IL2-STAT5 signaling, TNFα signaling via NF-κB, KRAS signaling up, and PI3K-AKT-mTOR signaling) relevant processes (**Figure 4A**). Further enrichment analysis using the KEGG also confirmed these results (**Figure 4B**).

Due to the notable variations in immune processes across different clusters, we quantified the infiltration levels of microenvironmental immune cells and immune checkpoint expression in high and low TLR3 expression groups. As shown in **Figure 4C**, the estimation of various immune cell type abundances through the use of TIMER algorithms. Compared to the low TLR3 expression group, the high TLR3 expression group presented higher levels of immune checkpoint protein expression and a greater abundance of immune cell infiltration. Additionally, Previous studies have shown that T cells can induce a series of immune responses and clear tumor cells by binding to major MHC molecules (30). Therefore, we investigated the correlation between TLR3 and five immunomodulatory factors (chemokines, immune inhibitor, immune stimulators, MHC, receptors). Our data showed that TLR3 had the strongest positive correlation with MHC molecules involved in antigen presentation (r = 0.52, *P* < 0.001) (**Figures 4D, E**).

### 3.3 TLR3 enhance the anticancer effects of sintilimab in LUAD

**Figure 5:**
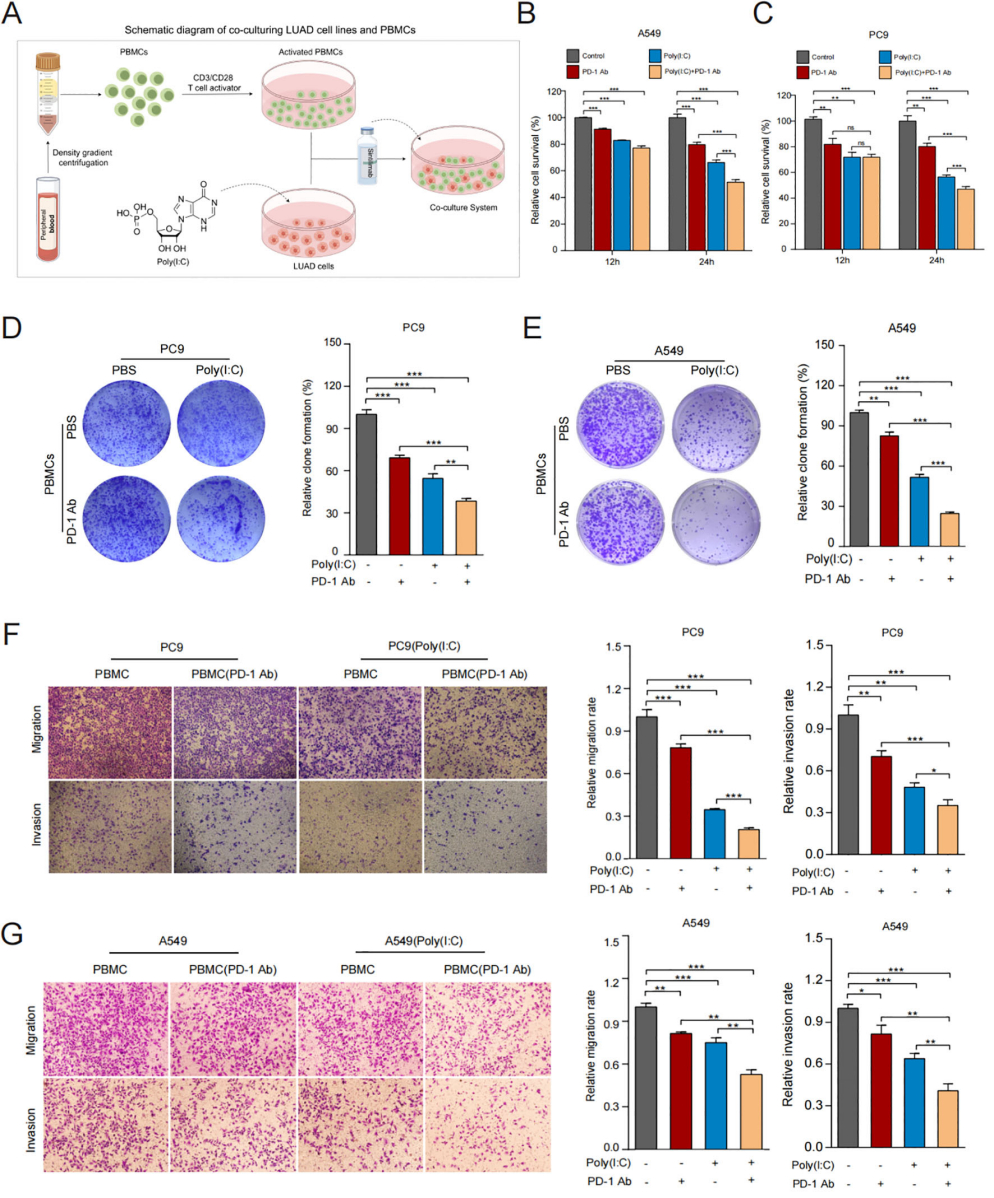
TLR3 promotes anti-tumor immune response *in vitro*. **(A)** Schematic diagram of co-culturing LUAD cell lines and PBMCs. **(B, C)** Cell viability of A549 and PC9 cells measured by CCK8 after co-culturing. **(D, E)** Proliferative ability of A549 and PC9 cells measured by colony formation assay after co-culturing. **(F, G)** Migration and invasion ability of A549 and PC9 cells measured by Transwell assay after co-culturing. Data shown as mean ± SD. *P<0.05, **P < 0.01, ***P < 0.001.

To examine how TLR3 influences the anticancer activity of PBMCs with or without the presence of ICIs, we established an LUAD cells/PBMCs co-culture system *in vitro*. The schematic diagram is shown in **Figure 5A**. The co-culture system of PBMCs and LUAD cells was supplemented with sintilimab. The results showed that LUAD cells treated with TLR3 agonists were more susceptible to activated PBMCs under PD1 inhibitor therapy, leading to inhibited cell proliferation (**Figures 5B, C**). The trend was further validated by colony formation assay (**Figures 5D, E**). Additionally, we found that PBMCs inhibited the migration and invasion potential of LUAD cells treated with TLR3 agonists, and the addition of PD1 inhibitors further enhanced this inhibitory effect (**Figures 5F, G**). These findings suggest that TLR3 agonists promote the lymphocytoxicity of PBMCs and elicit a more robust antitumor immune response in LUAD cells.

### 3.4 TLR3 regulates PD-L1 expression through NF-κB pathway

**Figure 6:**
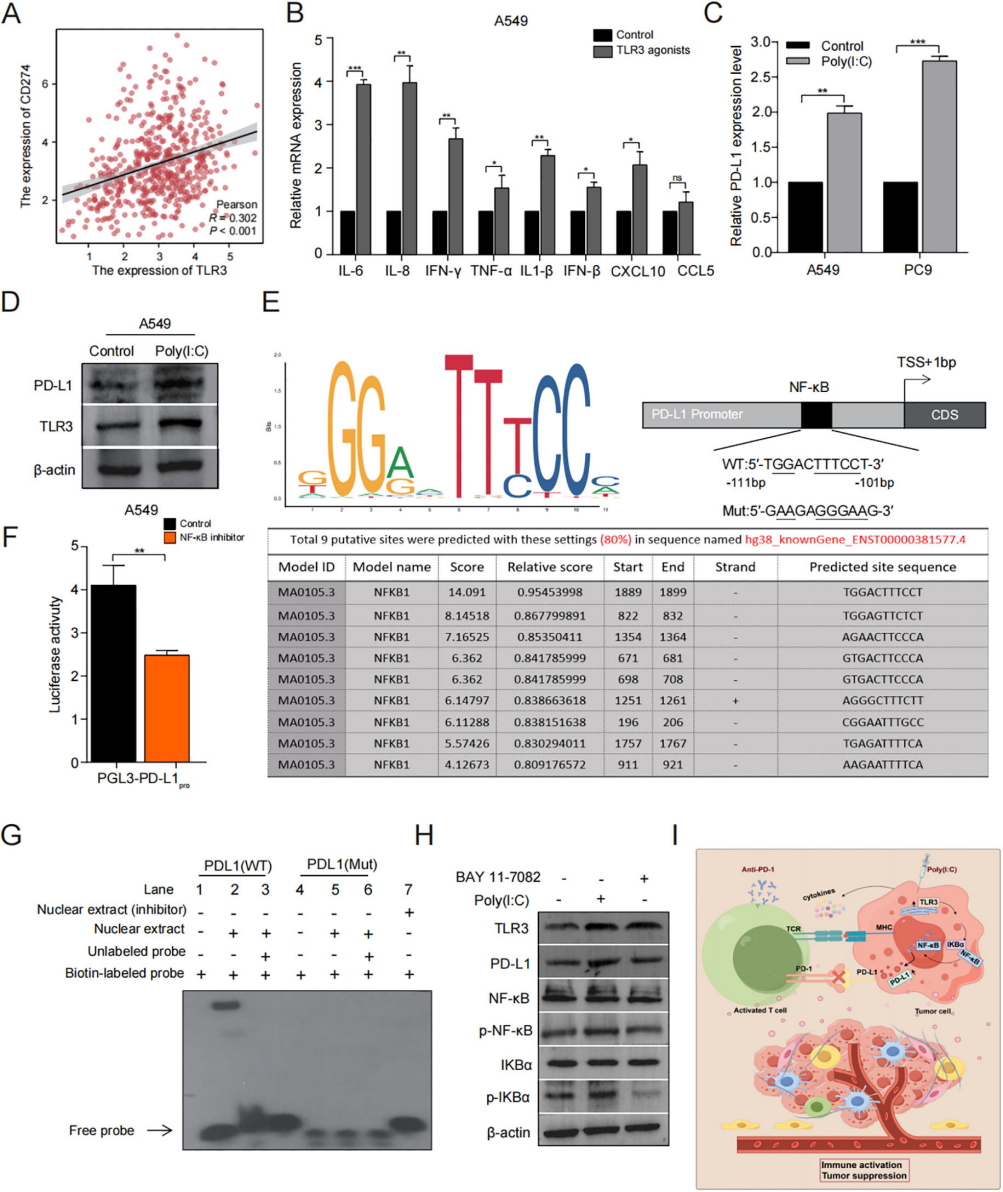
TLR3 regulates PD-L1 expression through NF-κB pathway. **(A)** Correlation between TLR3 and PD-L1 expression in the TCGA cohort. **(B)** The effect of TLR3 expression on cytokine release of A549 cells. **(C, D)** qRT-PCR and Western blotting analyses of PD-L1 regulated by TLR3. **(E)** JASPAR2024 prediction of the binding sequence of NF-κB to PD-L1. **(F)** Luciferase activity of pGL3-PD-L1pro in A549 cells treated with or without NF-κB inhibitor. **(G)** The binding activity of NF-κB to PD-L1 promoter identified by EMSA. **(H)** Western blotting analyses of the impact of TLR3 agonists and Bay-11 on NF-κB signaling. **(I)** Schematic diagram depicting the regulatory role of TLR3 in the TME immune landscapes of LUAD. Data shown as mean ± SD. *P < 0.05, **P < 0.01, ***P < 0.001, ns, no significance.

To explore the immune signaling pathways affected by TLR3, we reviewed the literature and found that NF-κB signaling essential for immune responses, inflammatory response and cancer therapy (31). Additionally, previous researchs indicate that elevated levels of PD-L1 expression and activation of tumor infiltrating lymphocytes (TILs) enhance the efficacy of PD-L1/PD-L1 immunotherapy (32). Based on correlation analysis utilizing the TCGA database revealed a positive relationship between the expression of PD-L1 and TLR3 (r = 0.302, *P* < 0.001) (**Figure 6A**). In addition, the treatment of LUAD cells with TLR3 agonists leads to a notable enhancement of cytokines expression compare to the control group (**Figure 6B**) and increase in PD-L1 expression (**Figures 6C, D**).

Based on the primary binding attributes of common transcription factors (TFs) as detailed in the JASPAR2024 database, NF-κB has been recognized as the most probable binder to PD-L1 (**Figure 6E**). Subsequently, we assessed how NF-κB influences the activity of PD-L1 promoter through luciferase assay and found that the promoter activity of PD-L1 decreased significantly when treated with NF-κB inhibitor (*P* < 0.001) (**Figure 6F**). Further analysis via EMSA showed that NF-κB can bind at the motif identified upstream of the PD-L1 (**Figure 6G**). These results demonstrate that NF-κB can regulate PD-L1 at the transcriptional level.

Next, we examined the expression levels of marker proteins associated with the NF-κB signaling pathway in A549 and PC9 cells with or without the addition of a TLR3 agonist (Poly(I:C)). This result was further validated by treatment with NF-κB inhibitor (BAY 11-7082). These results demonstrated that the level of TLR3, PD-L1, p-IκBα and p-NF-κB was significantly elevated in the Poly(I:C) treated group in comparison to the control group. However, after treatment with the addition of NF-κB inhibitor, these proteins were significantly inhibited (**Figure 6H**).

In conclusion, these findings indicate that TLR3 may regulate NF-κB signaling, thereby influencing the PD-L1 expression that could be pharmacologically targeted as an effective approach to sensitize patients with LUAD to ICIs (**Figure 6I**).

## 4 Discussion

Our study has revealed the pivotal role of TLR3 in regulating the immune landscape of LUAD, with particular emphasis on its potential to enhance the efficacy of immunotherapy. By analyzing multiple cohorts validation, we found that patients with higher levels of TLR3 demonstrated significantly improved survival, increased immune infiltration, and higher TMB, positioning TLR3 as a critical immune modulator in the tumor microenvironment.

Previous studies on TLR3 have primarily concentrated on its role in recognizing viral infections, mediating inflammation, and activating immune cells. For instance, TLR3 activation stimulates the production of various cytokines and chemokines via signal transduction pathways, including NF-κB and IRF3, thereby effectively counteracting viral invasion and regulating the homeostasis of the immune system (33, 34). As a crucial component of the immune system, the activation of the TLR3 receptor in dendritic cells significantly enhances the secretion efficiency of various cytokines, thereby effectively facilitating the activation process and functional differentiation of T lymphocytes (35, 36). However, recent studies have shown that the increased expression of endogenous TLR3 in tumor cells can improve the responses of CD4^+^ Th1 and CD8^+^ cytotoxic T cells (37, 38), and is used to improve immunotherapy by innate immunity in the TMEt (39, 40). Recently, increasing evidence has shown that combing TLR agonists with other therapies can effectively eliminate tumors (41). For example, combining Poly(I:C) with paclitaxel successfully increases tumor cytotoxicity in drug-resistant colon cancer cells through IFN-β secretion (42). Similarly, the combination of a TLR3 agonist with sorafenib can hinder the advancement of hepatocellular carcinoma through the activation of NK and CD8^+^ T cells (43). Additionally, a multi-center study also reported improved outcomes using a combined therapy of Poly(I:C), radiotherapy and temozolomide for glioblastoma treatment (44).

Our data show that TLR3 activation may enhance the effectiveness of PD1 inhibitors by amplifying the response within the TME. Given that ICIs have significantly prolonged the OS of patients with various cancers, including lung adenocarcinoma (45–47). However, many patients exhibit limited or no response to ICI therapies (3, 48, 49). Studies have shown that after treatment with anti-PD1 antibodies, the survival rate of patients with high expression of PD-L1 has been significantly improved (17–20). But PD-L1 expression levels vary greatly between individuals (50, 51). Therefore, PD-L1 expression serves as a critical determinant for evaluating clinical responses to anti- PD1 therapy (52, 53).

Multiple mechanisms are involved in the regulation of PD-L1 expression, with the regulation by transcription factors such as STAT3, AP-1, HIF-1α and NF-κB being included (54–58), as well as the regulation of histones and post-translational modifications (ubiquitination and acetylation) (59). Several studies have shown that TLR3 can activate nuclear NF-κB (60), which leads to the production of type I interferons and chemokines (61, 62). It is reported that the PARP1 inhibitor Olaparib can promotes the binding of the transcription factor nuclear phosphoprotein (NPM1) to the PD-L1 promoter in triple-negative breast cancer cells,thereby activating PD-L1 transcription (63). Which boosts the effectiveness of anti-PD-1 treatment. Similarly, our study observed a greater presence of CD8+T cells, CTLs, and NK cells in LUAD patients with elevated TLR3 expression, and to enhance the sensitivity of immune-tolerant LUAD to immunotherapy by regulating PD-L1 expression via the NF-κB pathway.

While these models provide valuable mechanistic insights, a limitation of our study is the reliance on *in vitro* and in silico data to define the relationship between TLR3 and immunotherapy response. Our future research will involve clinical validation in larger cohorts or prospective trials to confirm the utility of TLR3 as a biomarker or therapeutic target. Furthermore, we aim to elucidate the complex tumor microenvironment and associated mechanisms *in vivo*, particularly in response to combined immunotherapy, through experiments conducted in animal models.

## 5 Conclusion

In conclusion, this study offers a thorough analysis of the role of TLR3 in LUAD, demonstrating its potential to improve immunotherapy outcomes. By enhancing immune infiltration, TMB, and PD-L1 expression via the NF-κB pathway, TLR3 functions as both a biomarker for prognosis and a prospective therapeutic target.

## Data Availability

The original contributions presented in the study are included in the article/**Supplementary Material**. Further inquiries can be directed to the corresponding author.
