# Supplementary material for: Toll-like receptor 3 activation enhances antitumor immune response in lung adenocarcinoma through NF-κB signaling pathway
Source: Front Immunol. 2025 May 8;16:1585747. doi: 10.3389/fimmu.2025.1585747 (PMC12095255; doi:10.3389/fimmu.2025.1585747)
Supplement: Supplementary file 1 [file Table1.docx]

| Gene |  | Sequence (5'->3') |
| --- | --- | --- |
| PD-L1 | Forward primer | TGGCATTTGCTGAACGCATTT |
|  | Reverse primer | TGCAGCCAGGTCTAATTGTTTT |
| IL-6 | Forward primer | ACTCACCTCTTCAGAACGAATTG |
|  | Reverse primer | CCATCTTTGGAAGGTTCAGGTTG |
| IFN-γ | Forward primer | TCGGTAACTGACTTGAATGTCCA |
|  | Reverse primer | TCGCTTCCCTGTTTTAGCTGC |
| TNF-α | Forward primer | CCTCTCTCTAATCAGCCCTCTG |
|  | Reverse primer | GAGGACCTGGGAGTAGATGAG |
| IL-1β | Forward primer | ATGATGGCTTATTACAGTGGCAA |
|  | Reverse primer | GTCGGAGATTCGTAGCTGGA |
| IFN-β | Forward primer | ATGACCAACAAGTGTCTCCTCC |
|  | Reverse primer | GGAATCCAAGCAAGTTGTAGCTC |
| CXCL10 | Forward primer | GTGGCATTCAAGGAGTACCTC |
|  | Reverse primer | TGATGGCCTTCGATTCTGGATT |
| CCL5 | Forward primer | CCAGCAGTCGTCTTTGTCAC |
|  | Reverse primer | CTCTGGGTTGGCACACACTT |
| IL-8 | Forward primer | TTTTGCCAAGGAGTGCTAAAGA |
|  | Reverse primer | AACCCTCTGCACCCAGTTTTC |

**Supplementary Table 1**
