# Supplementary material for: Toll-like receptor 3 activation enhances antitumor immune response in lung adenocarcinoma through NF-κB signaling pathway
Source: Front Immunol. 2025 May 8;16:1585747. doi: 10.3389/fimmu.2025.1585747 (PMC12095255; doi:10.3389/fimmu.2025.1585747)
Supplement: Supplementary file 2 [file DataSheet1.zip › Original data/Western Blot original image.docx]

**Supplementary Information**


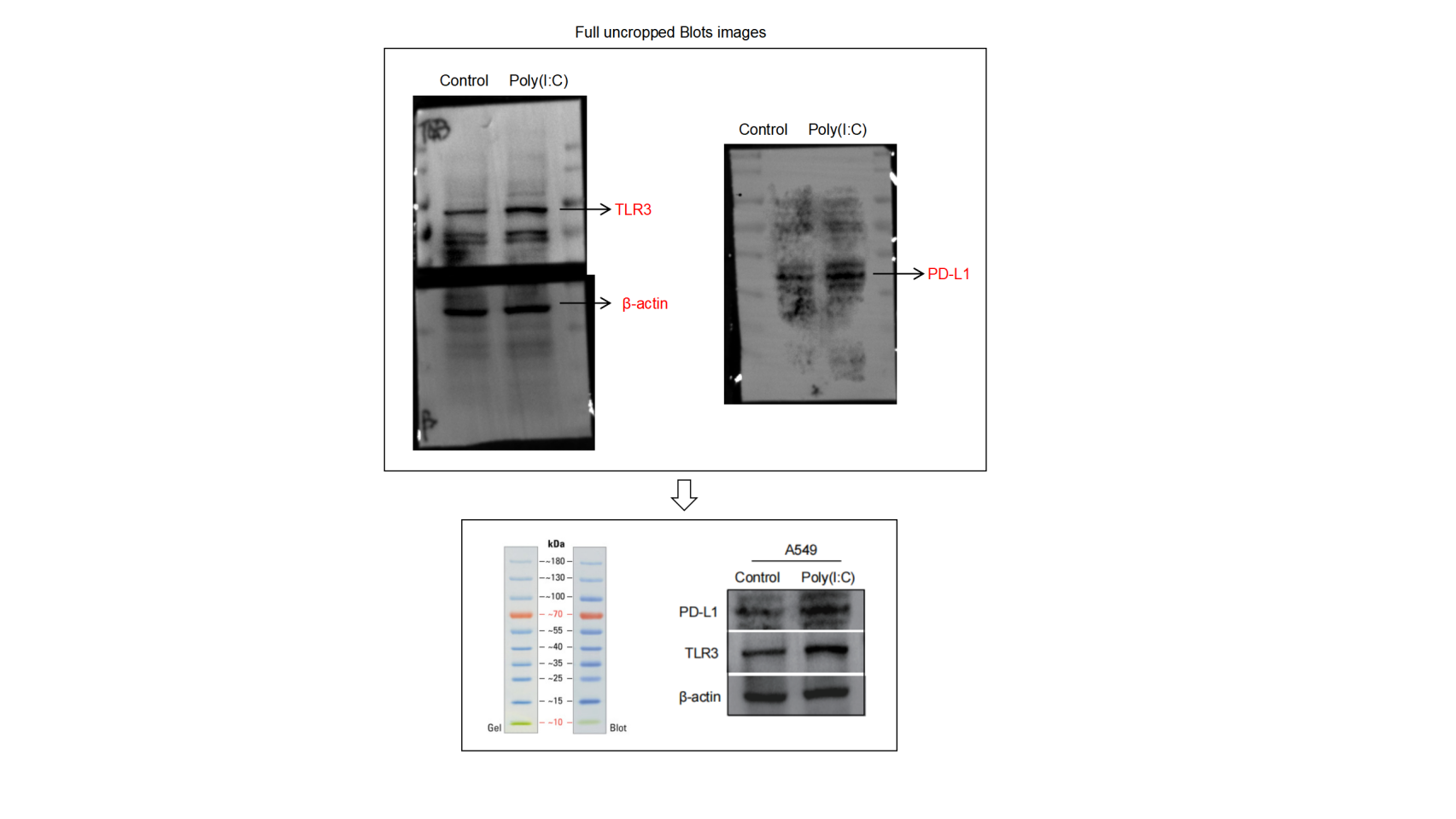


**Figure 6D** The original EMSA blot image (up).Western blotting analyses of PD-L1 regulated by TLR3


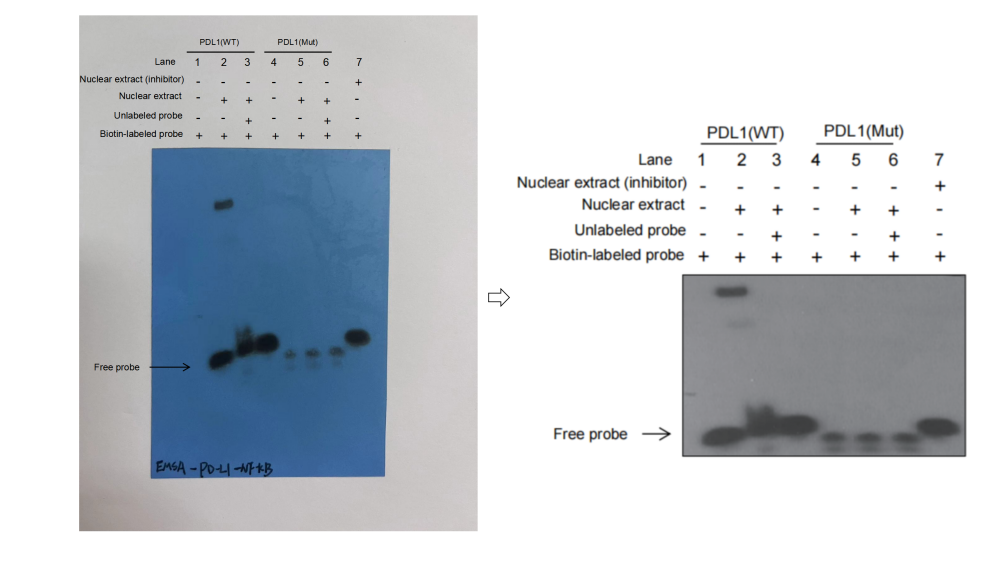


**Figure 6G** The original EMSA film image (left). The binding activity of NF-κB to PD-L1 promoter identified by EMSA.


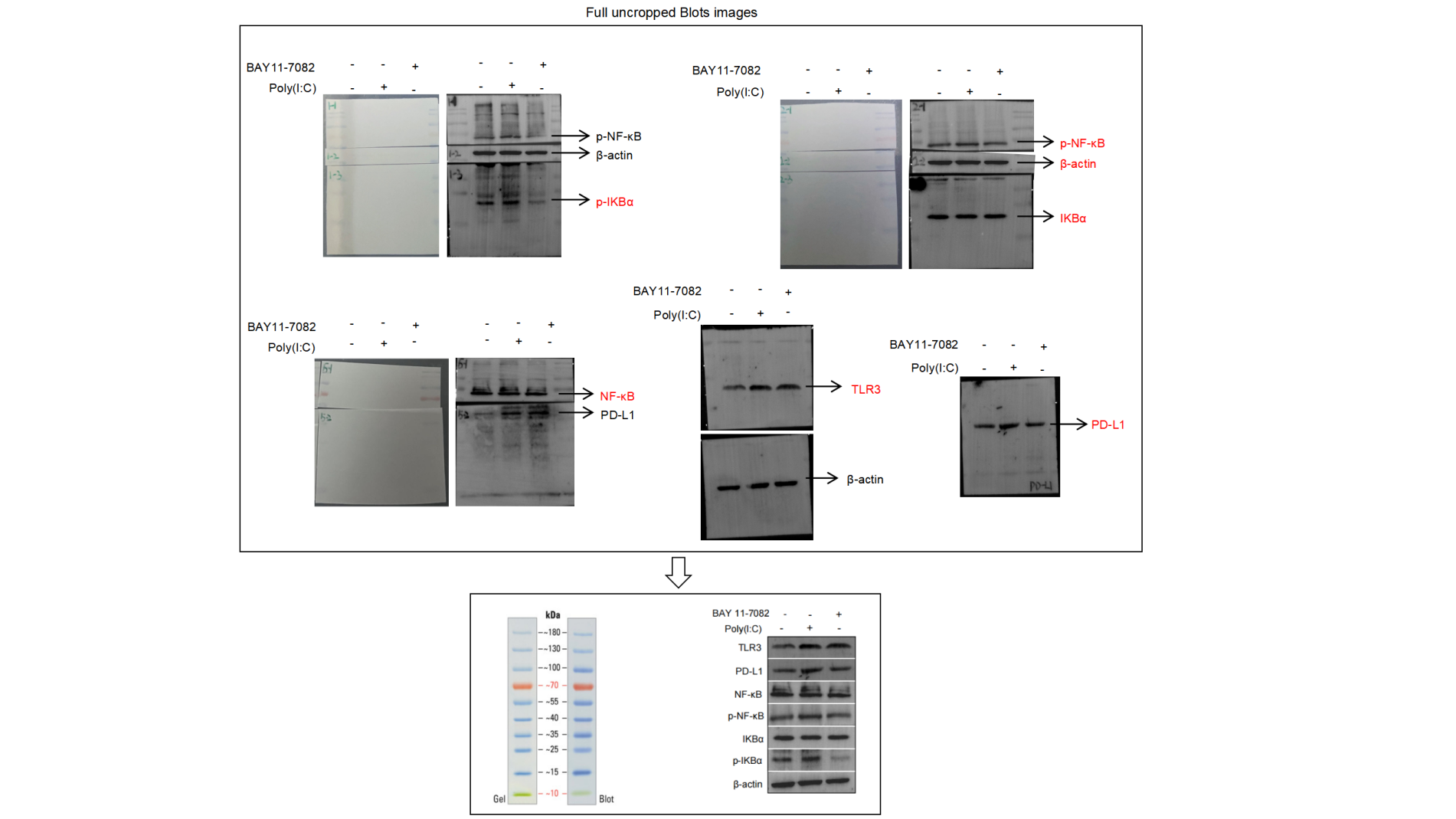


**Figure 6H** The original EMSA blot image (up). Western blotting analyses of the impact of TLR3 agonists and Bay11-7082 on NF-κB signaling.
