# Supplementary figures and images for: Toll-like receptor 3 activation enhances antitumor immune response in lung adenocarcinoma through NF-κB signaling pathway
Source: Front Immunol. 2025 May 8;16:1585747. doi: 10.3389/fimmu.2025.1585747 (PMC12095255; doi:10.3389/fimmu.2025.1585747)

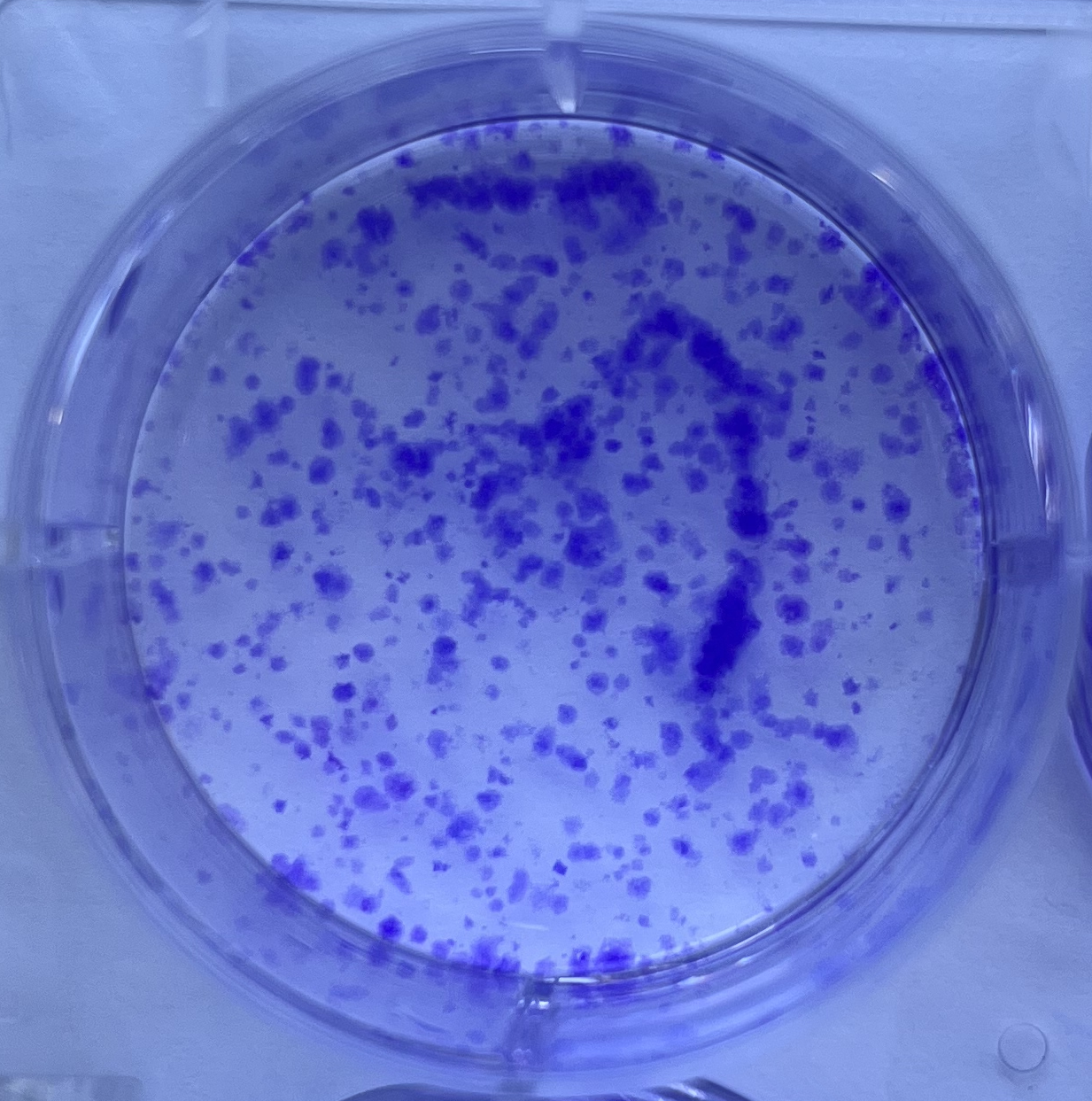

Supplement: Supplementary file 2 [file DataSheet1.zip › Original data/Fig 5D_PC9(Poly)+PBMC(Sintilimab).jpg]

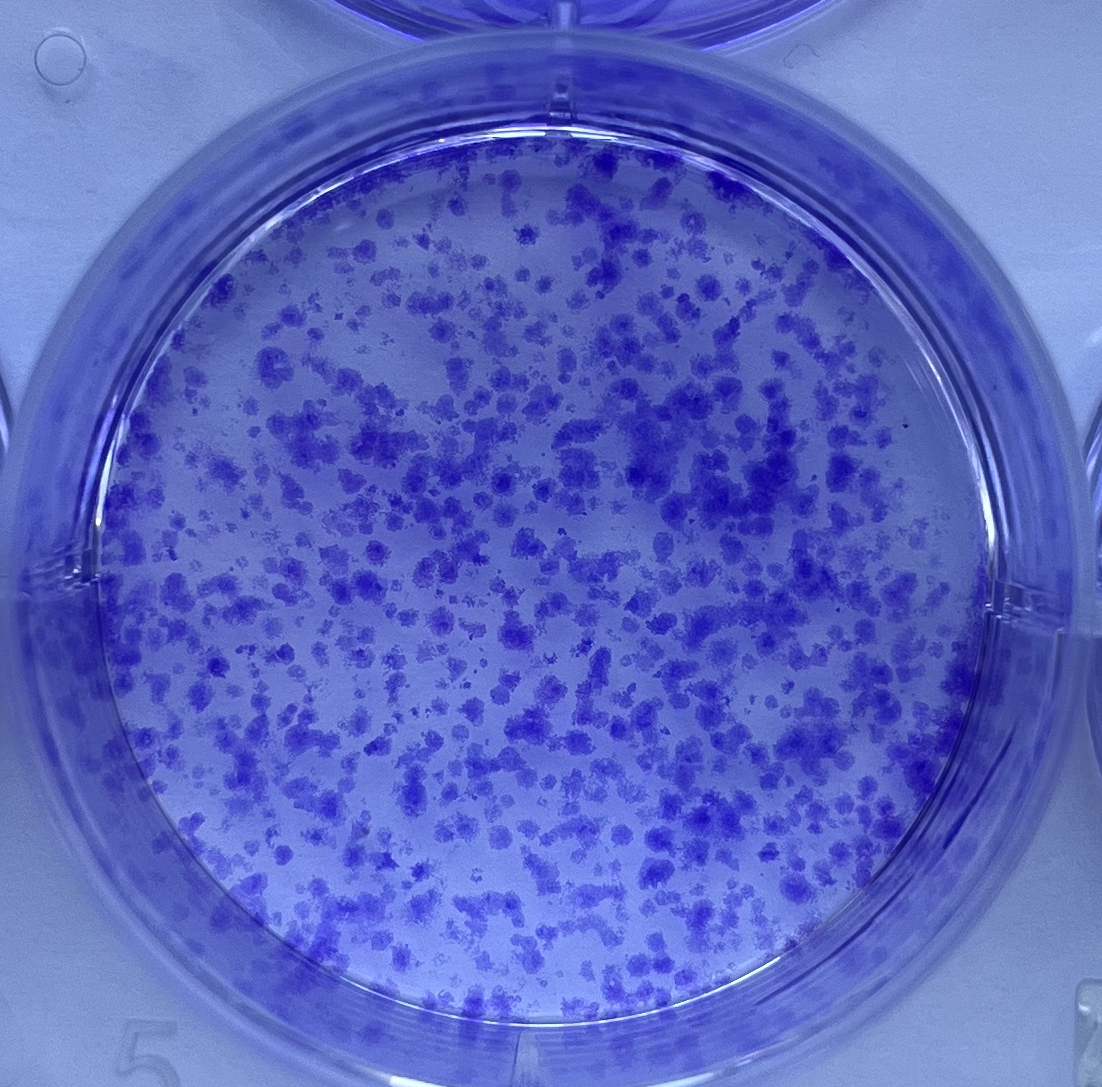

Supplement: Supplementary file 2 [file DataSheet1.zip › Original data/Fig 5D_PC9(Poly)+PBMC.jpg]

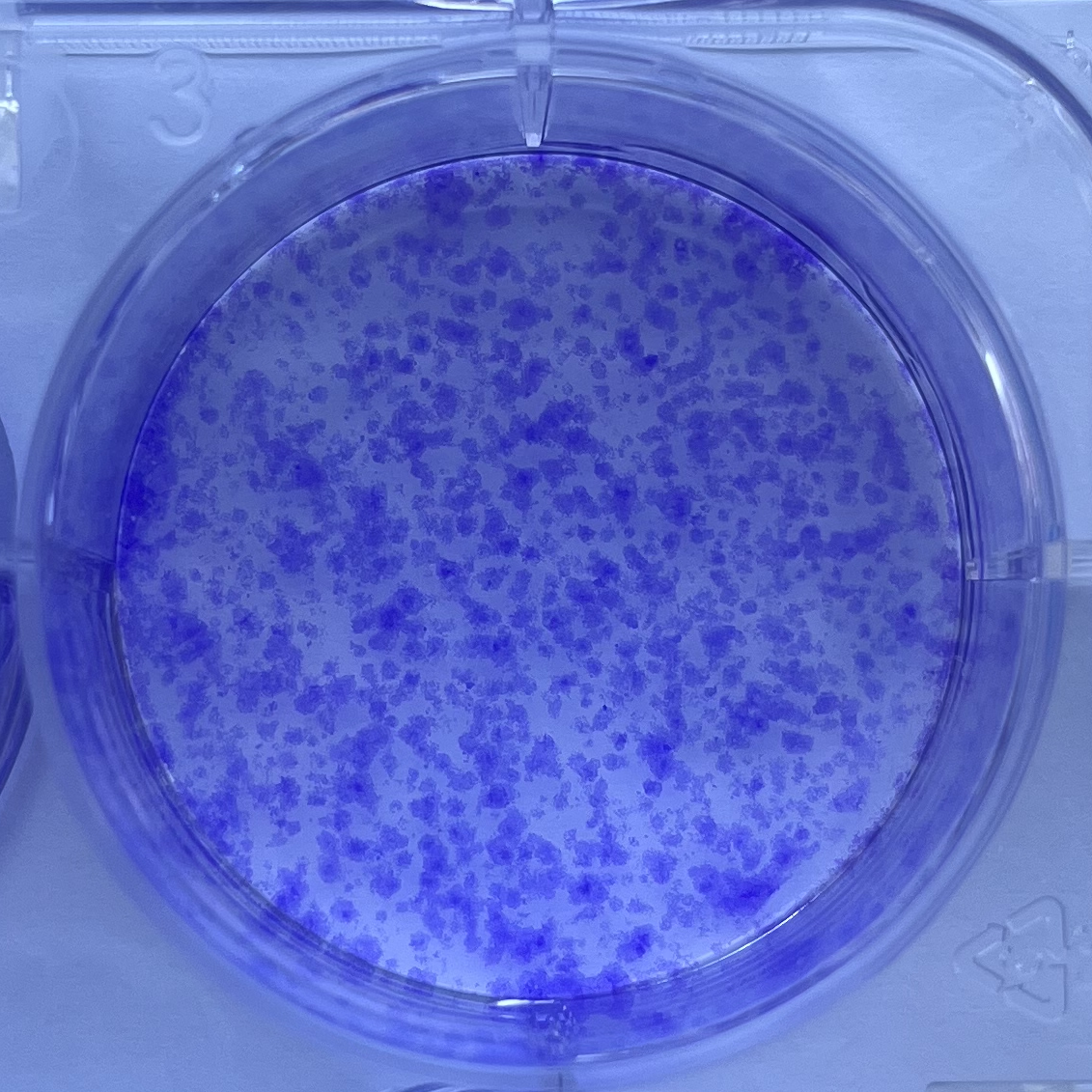

Supplement: Supplementary file 2 [file DataSheet1.zip › Original data/Fig 5D_PC9+PBMC(Sintilimab).jpg]

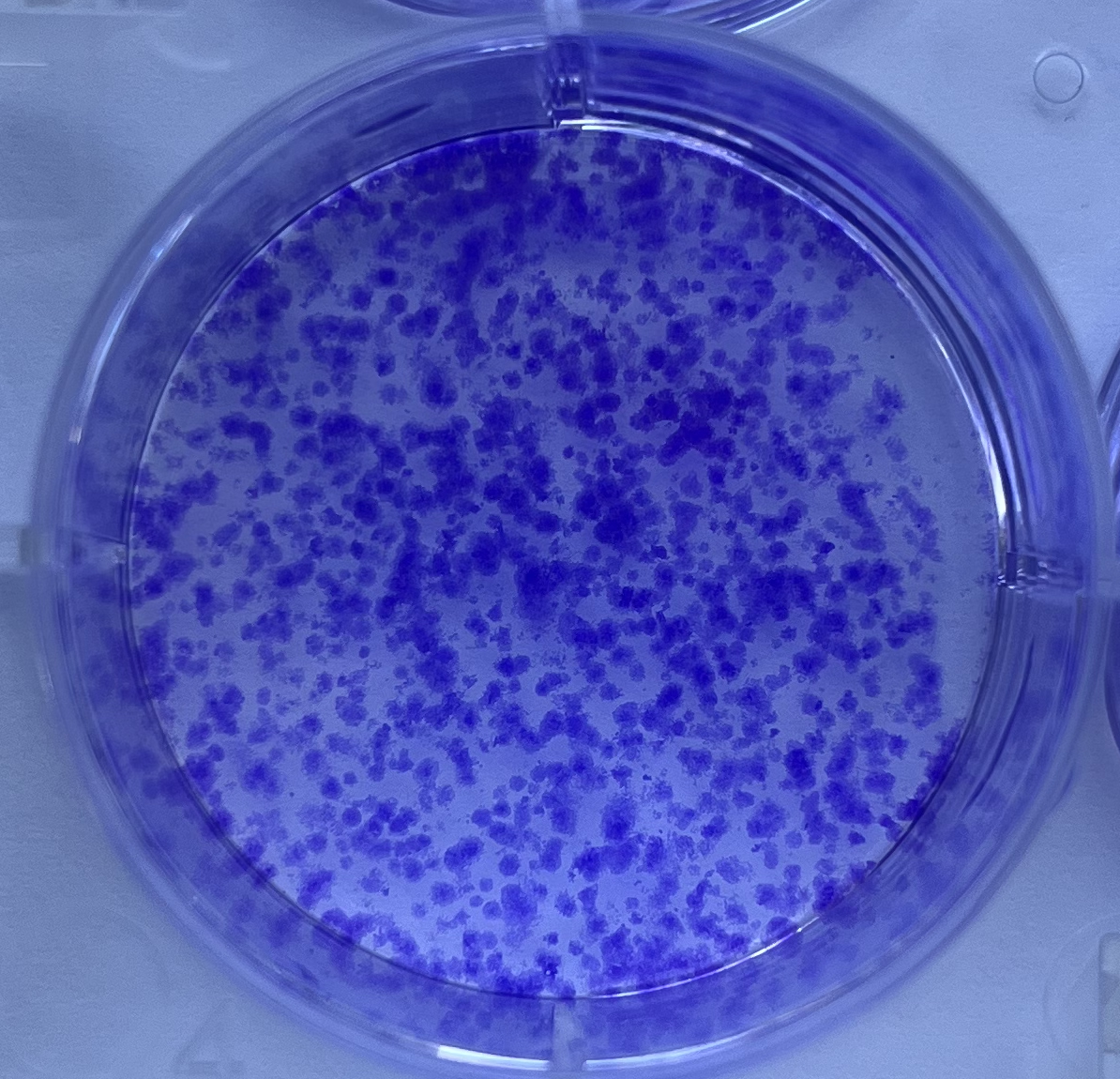

Supplement: Supplementary file 2 [file DataSheet1.zip › Original data/Fig 5D_PC9+PBMC.jpg]

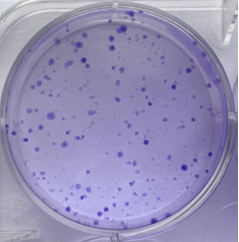

Supplement: Supplementary file 2 [file DataSheet1.zip › Original data/Fig 5E_A549(Poly)+PBMC(Sintilimab).tif]

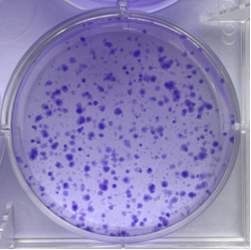

Supplement: Supplementary file 2 [file DataSheet1.zip › Original data/Fig 5E_A549(Poly)+PBMC.tif]

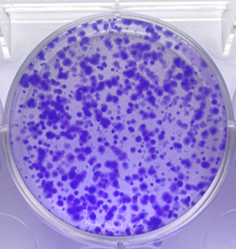

Supplement: Supplementary file 2 [file DataSheet1.zip › Original data/Fig 5E_A549+PBMC(Sintilimab).tif]

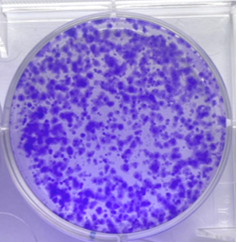

Supplement: Supplementary file 2 [file DataSheet1.zip › Original data/Fig 5E_A549+PBMC.tif]

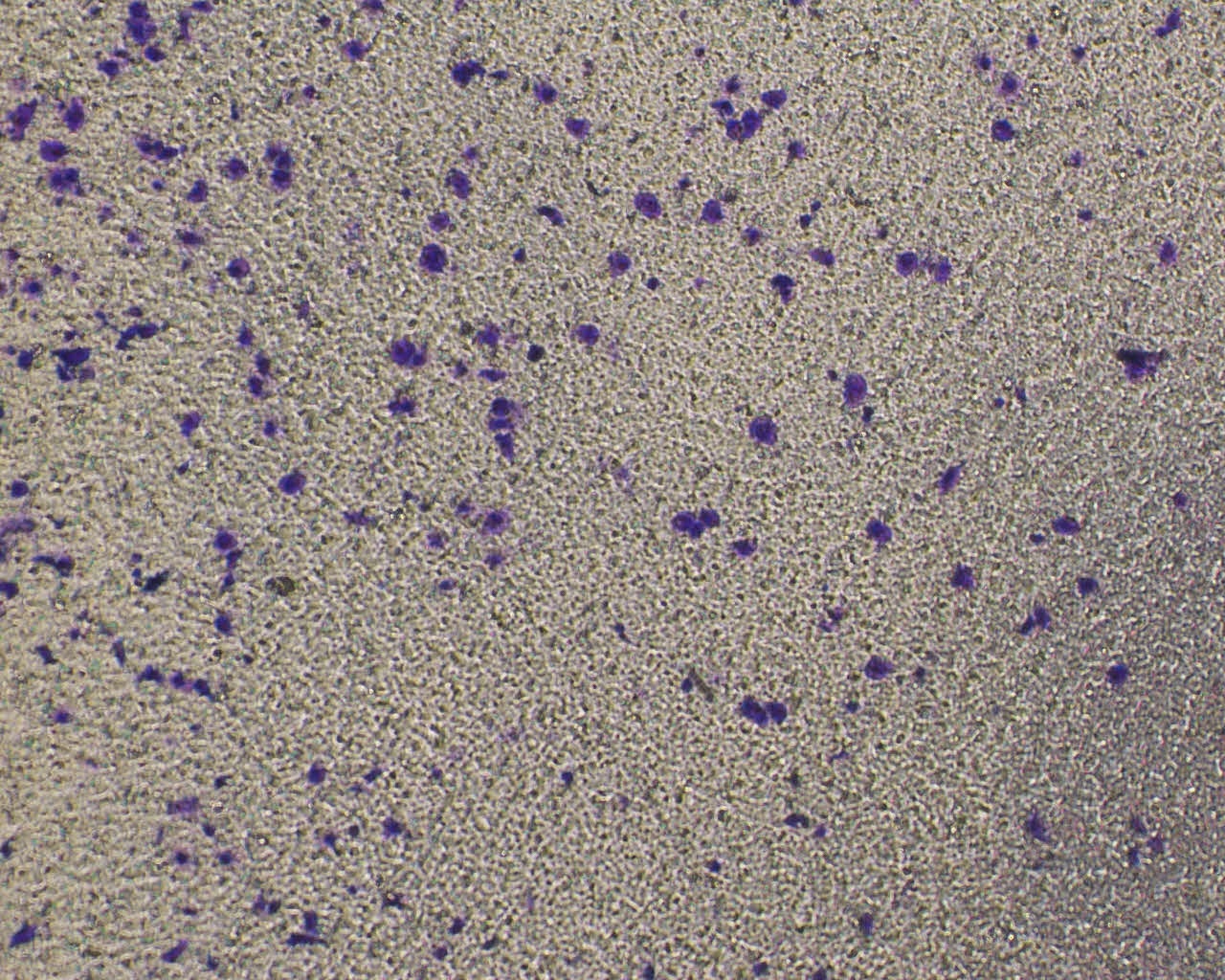

Supplement: Supplementary file 2 [file DataSheet1.zip › Original data/Fig 5F_PC9(Poly)+PBMC(Sintilimab)_Invasion.jpg]

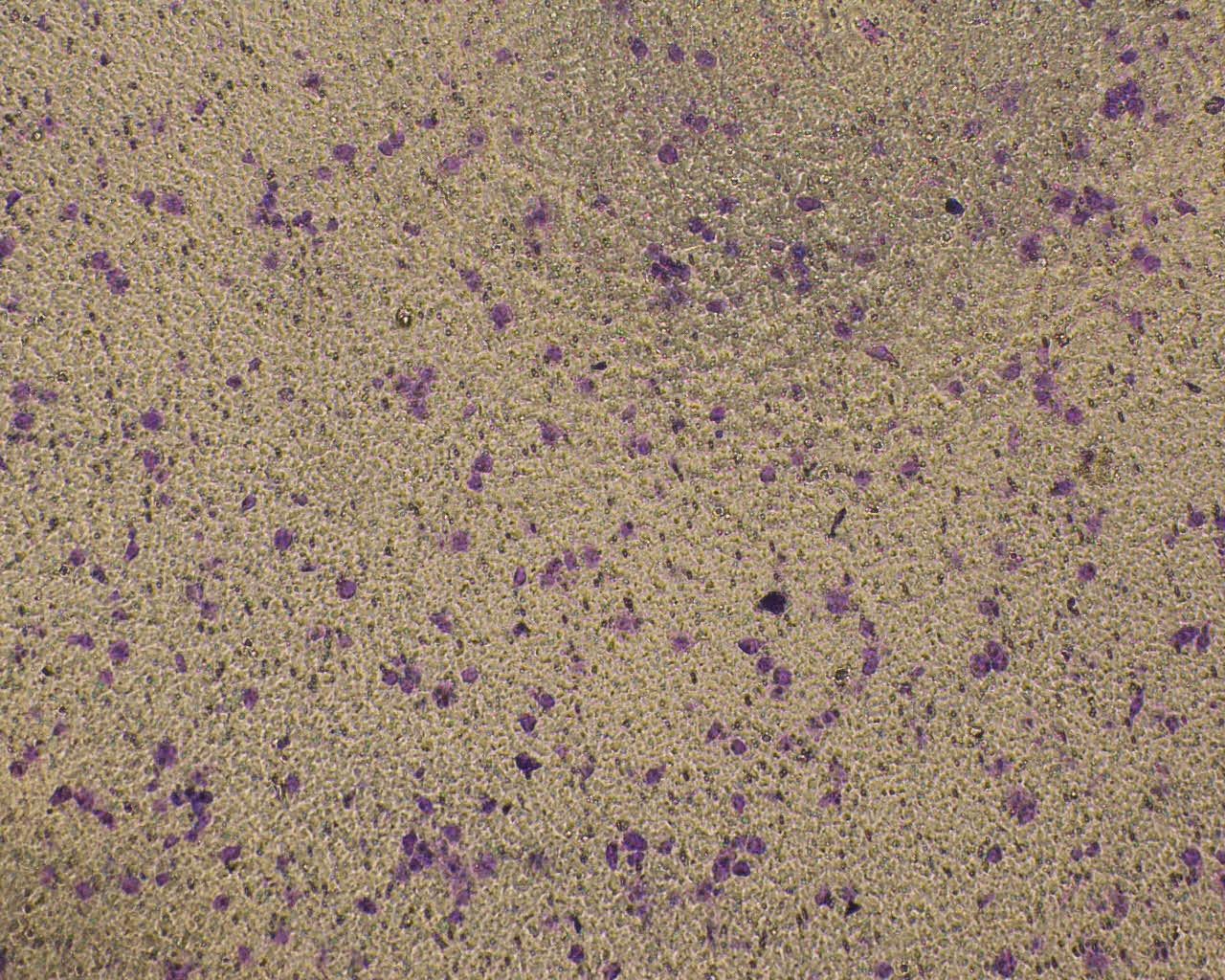

Supplement: Supplementary file 2 [file DataSheet1.zip › Original data/Fig 5F_PC9(Poly)+PBMC_Invasion.jpg]

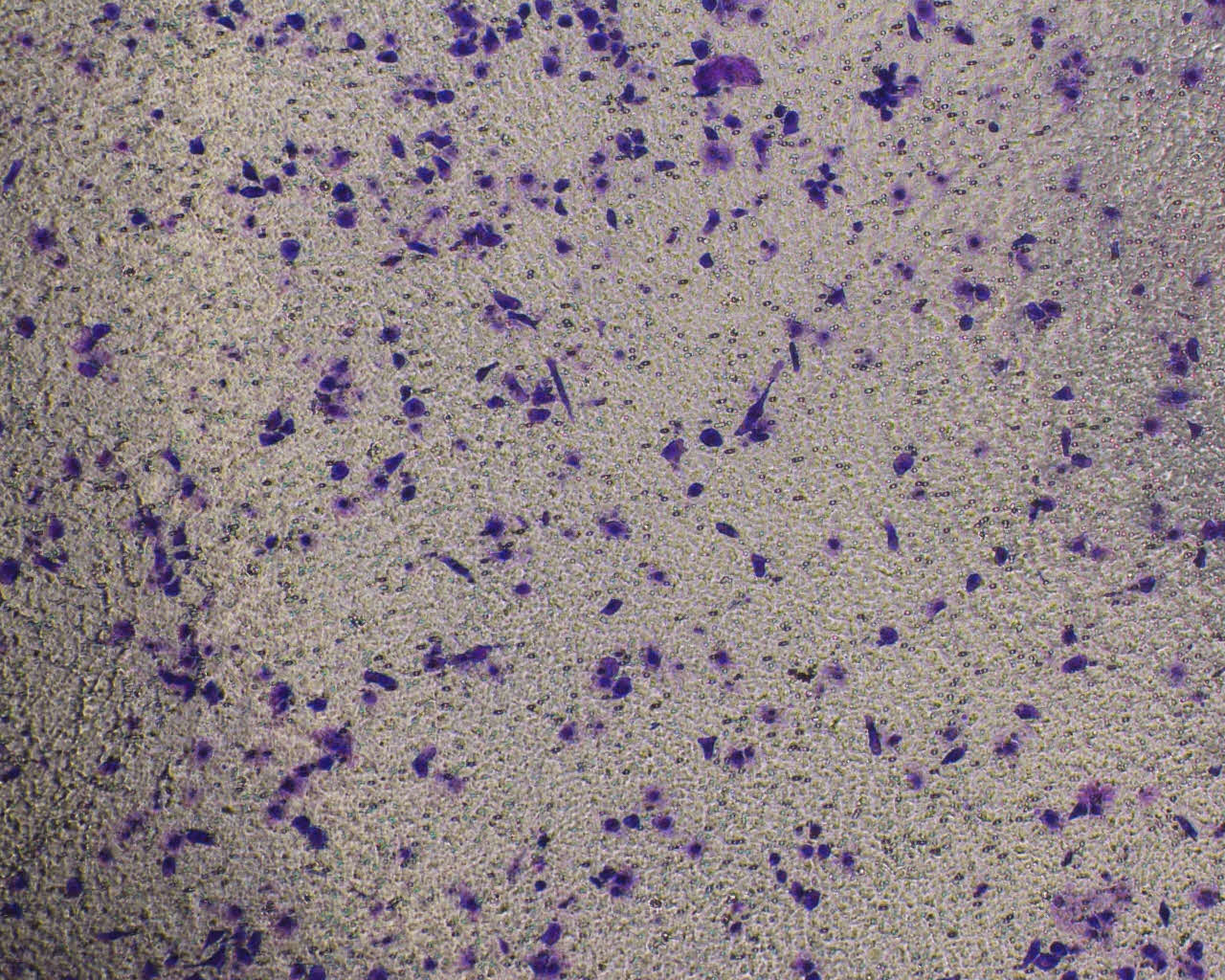

Supplement: Supplementary file 2 [file DataSheet1.zip › Original data/Fig 5F_PC9+PBMC(Sintilimab)_Invasion.jpg]

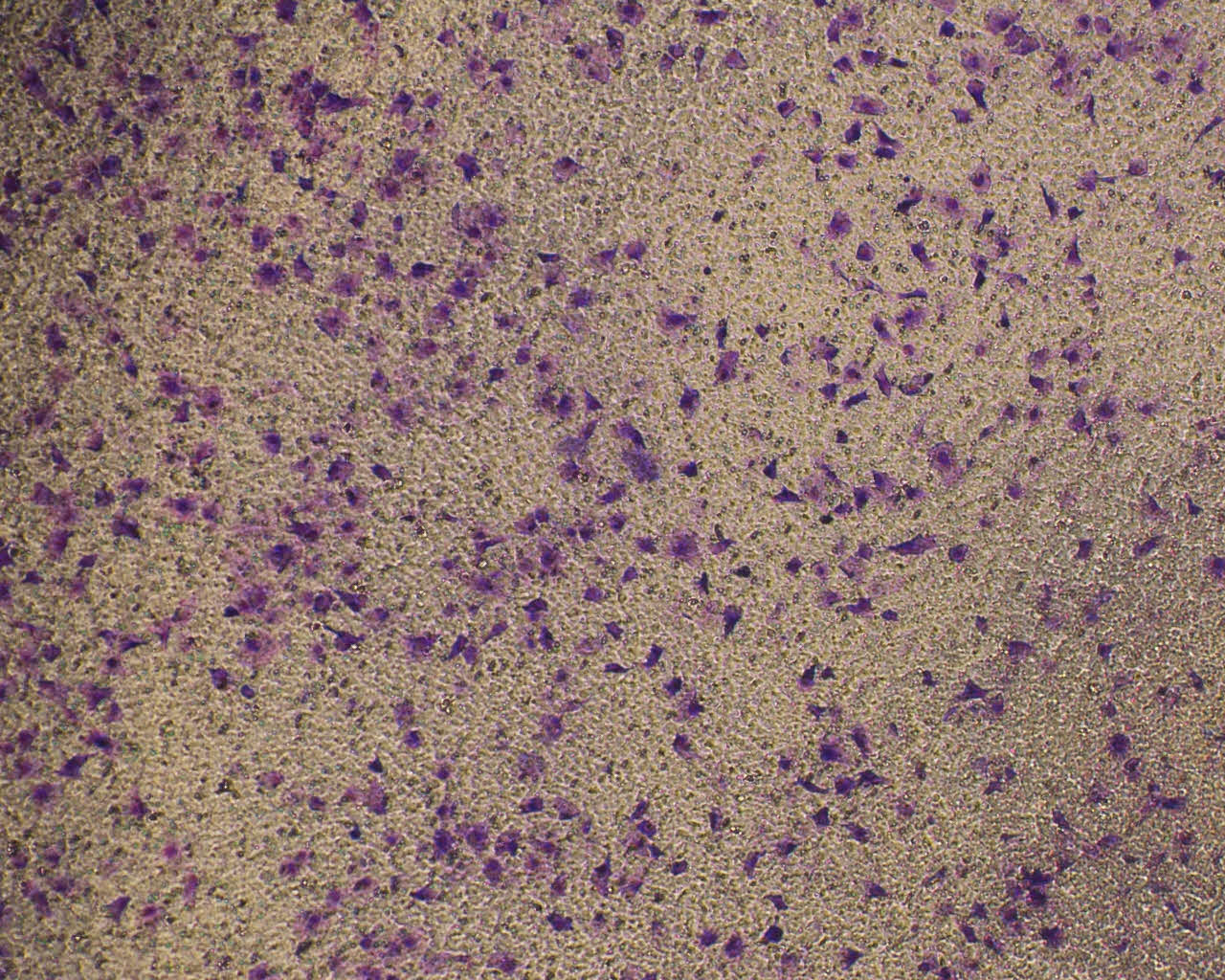

Supplement: Supplementary file 2 [file DataSheet1.zip › Original data/Fig 5F_PC9+PBMC_Invasion.jpg]

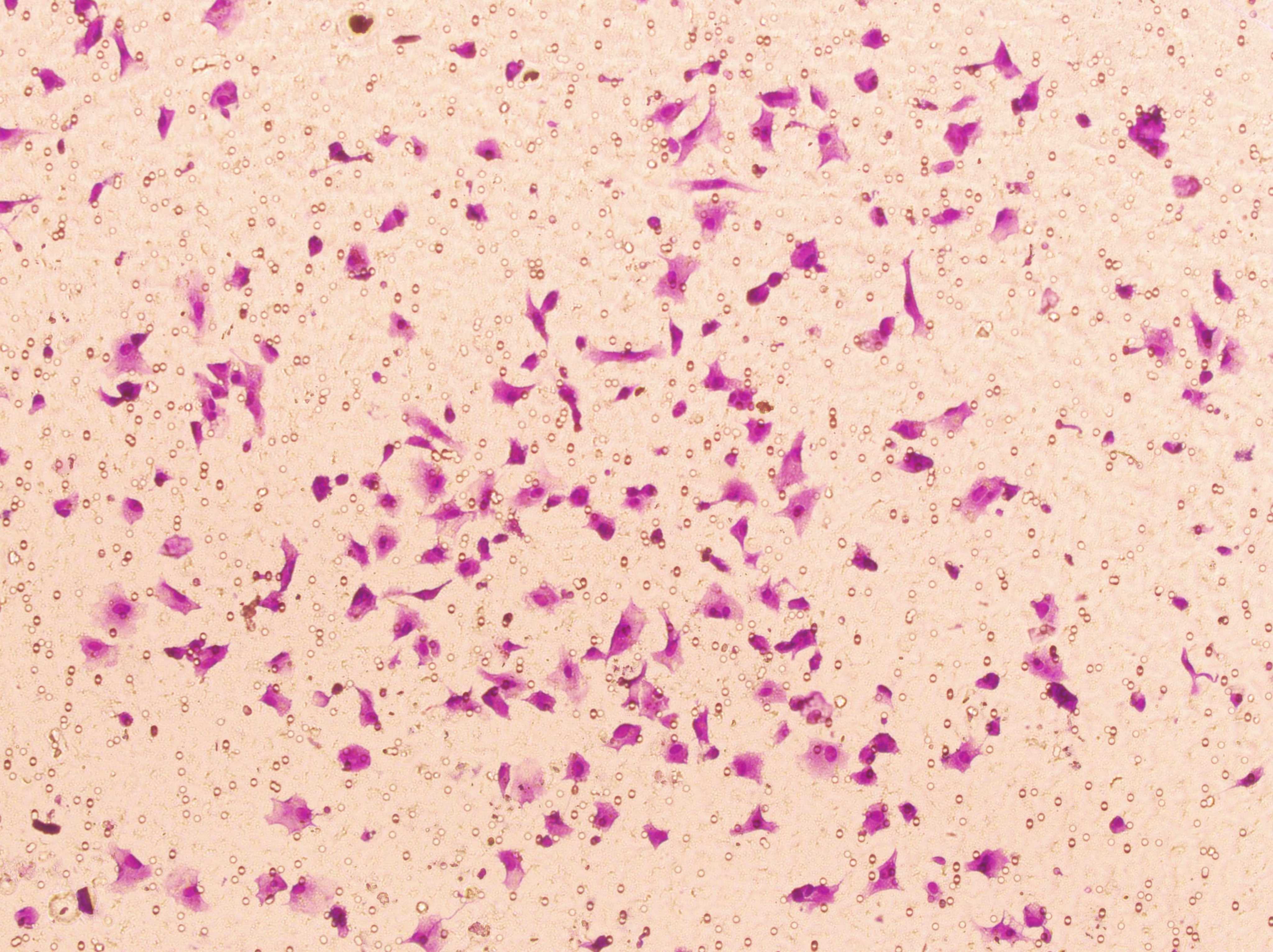

Supplement: Supplementary file 2 [file DataSheet1.zip › Original data/Fig 5G_A549(Poly)+PBMC(Sintilimab)_Invasion.jpg]

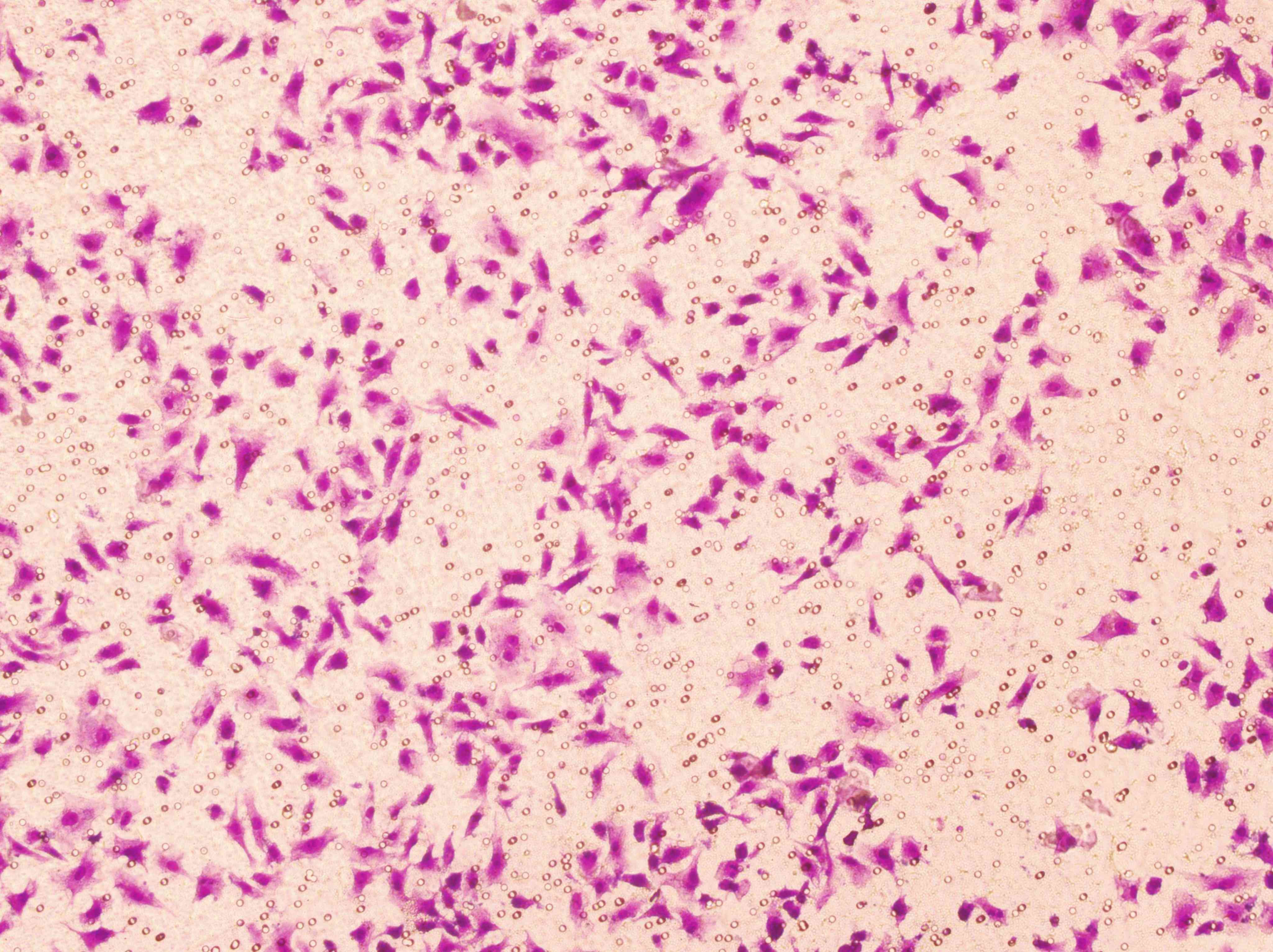

Supplement: Supplementary file 2 [file DataSheet1.zip › Original data/Fig 5G_A549(Poly)+PBMC(Sintilimab)_Migration.jpg]

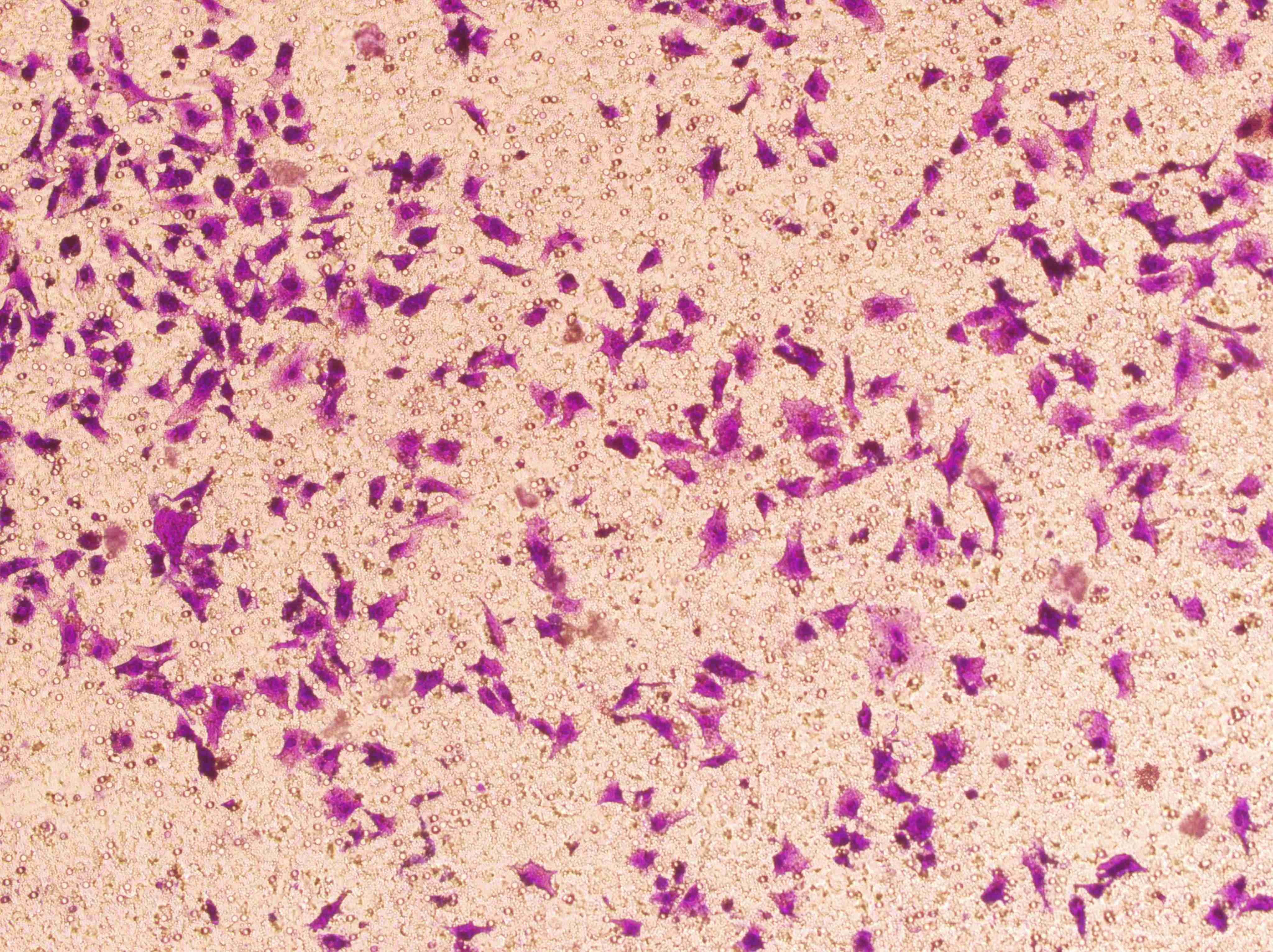

Supplement: Supplementary file 2 [file DataSheet1.zip › Original data/Fig 5G_A549(Poly)+PBMC_Invasion.jpg]

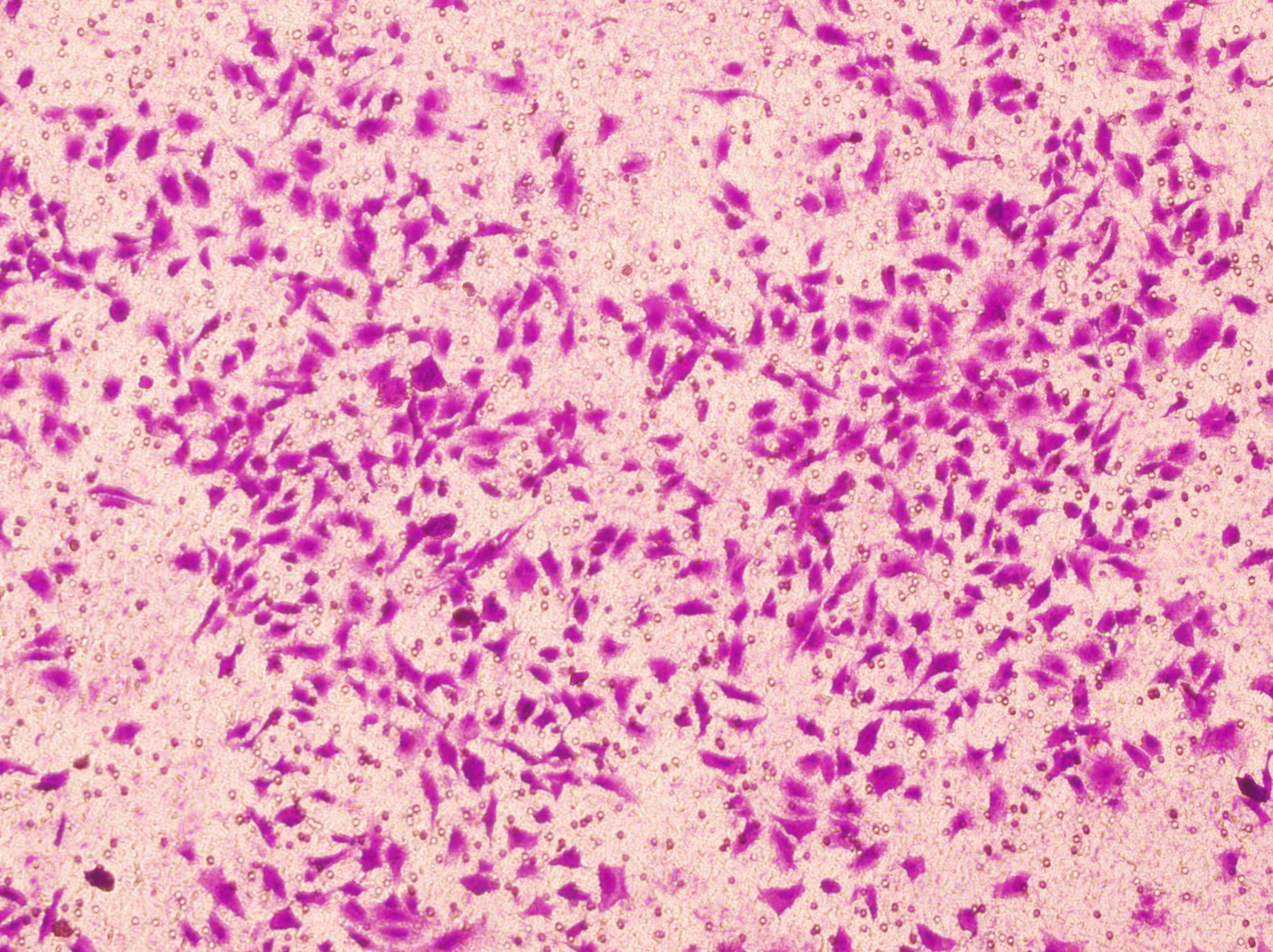

Supplement: Supplementary file 2 [file DataSheet1.zip › Original data/Fig 5G_A549(Poly)+PBMC_Migration.jpg]

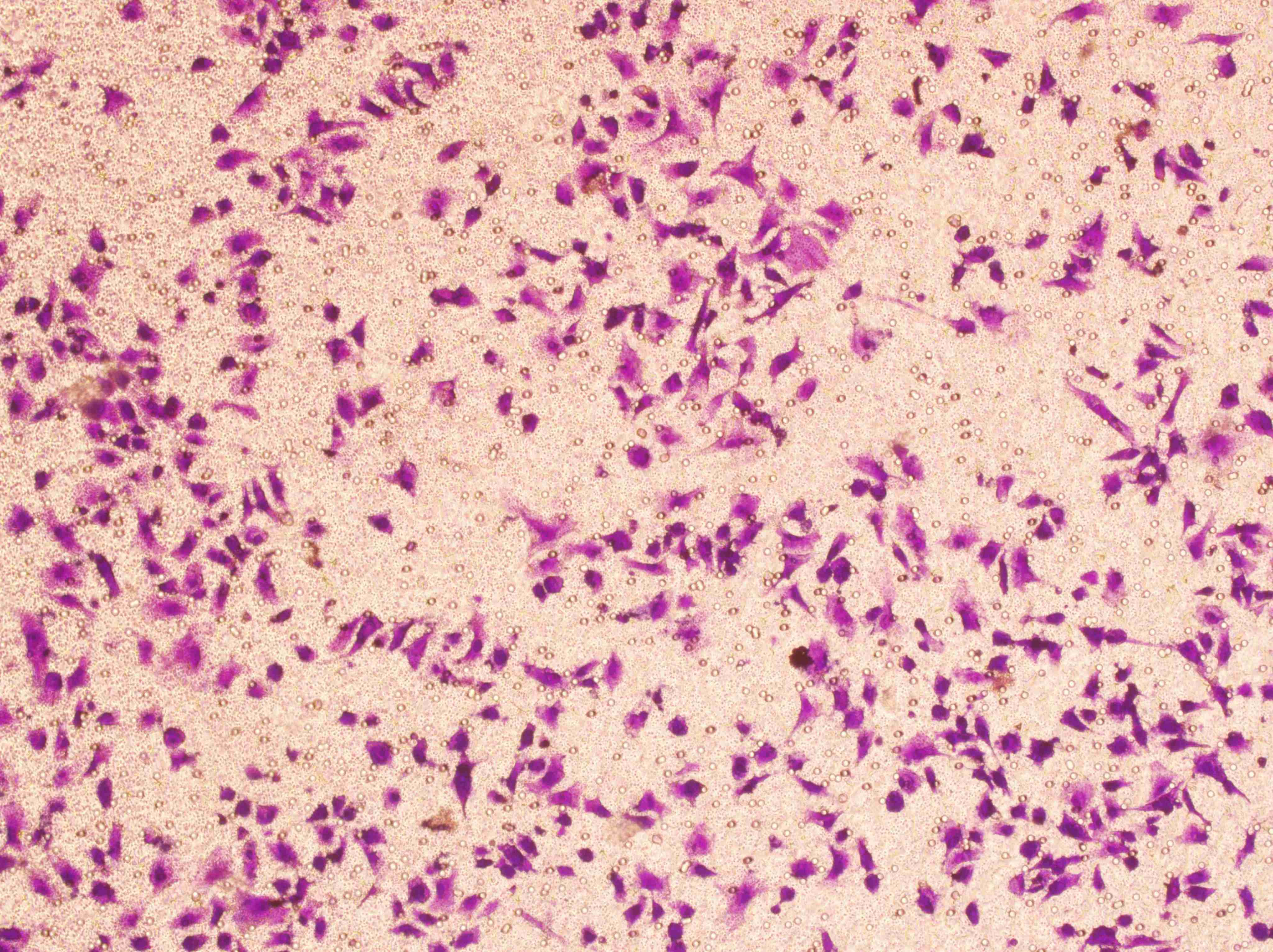

Supplement: Supplementary file 2 [file DataSheet1.zip › Original data/Fig 5G_A549+PBMC(Sintilimab)_Invasion.jpg]

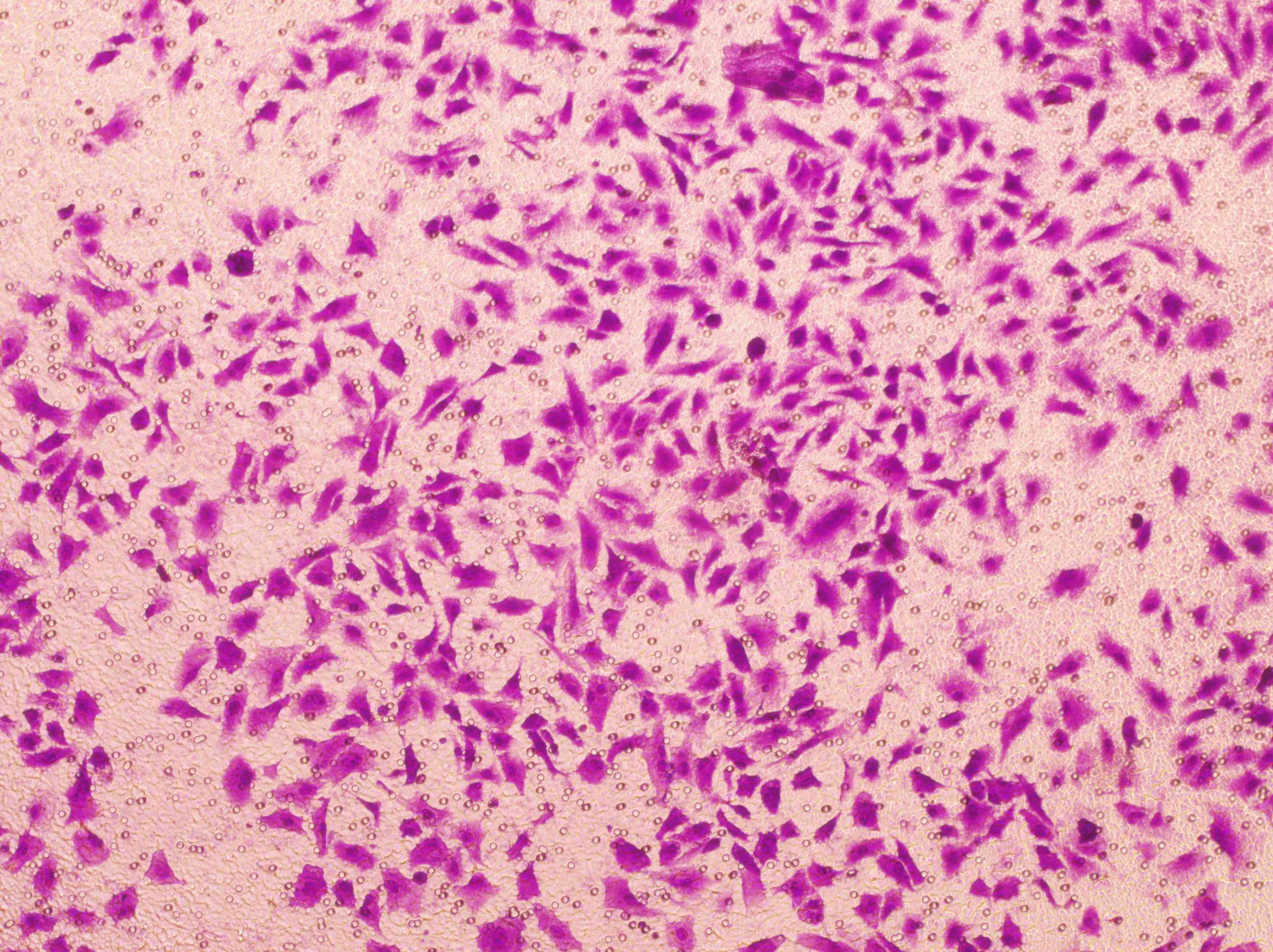

Supplement: Supplementary file 2 [file DataSheet1.zip › Original data/Fig 5G_A549+PBMC(Sintilimab)_Migration.jpg]

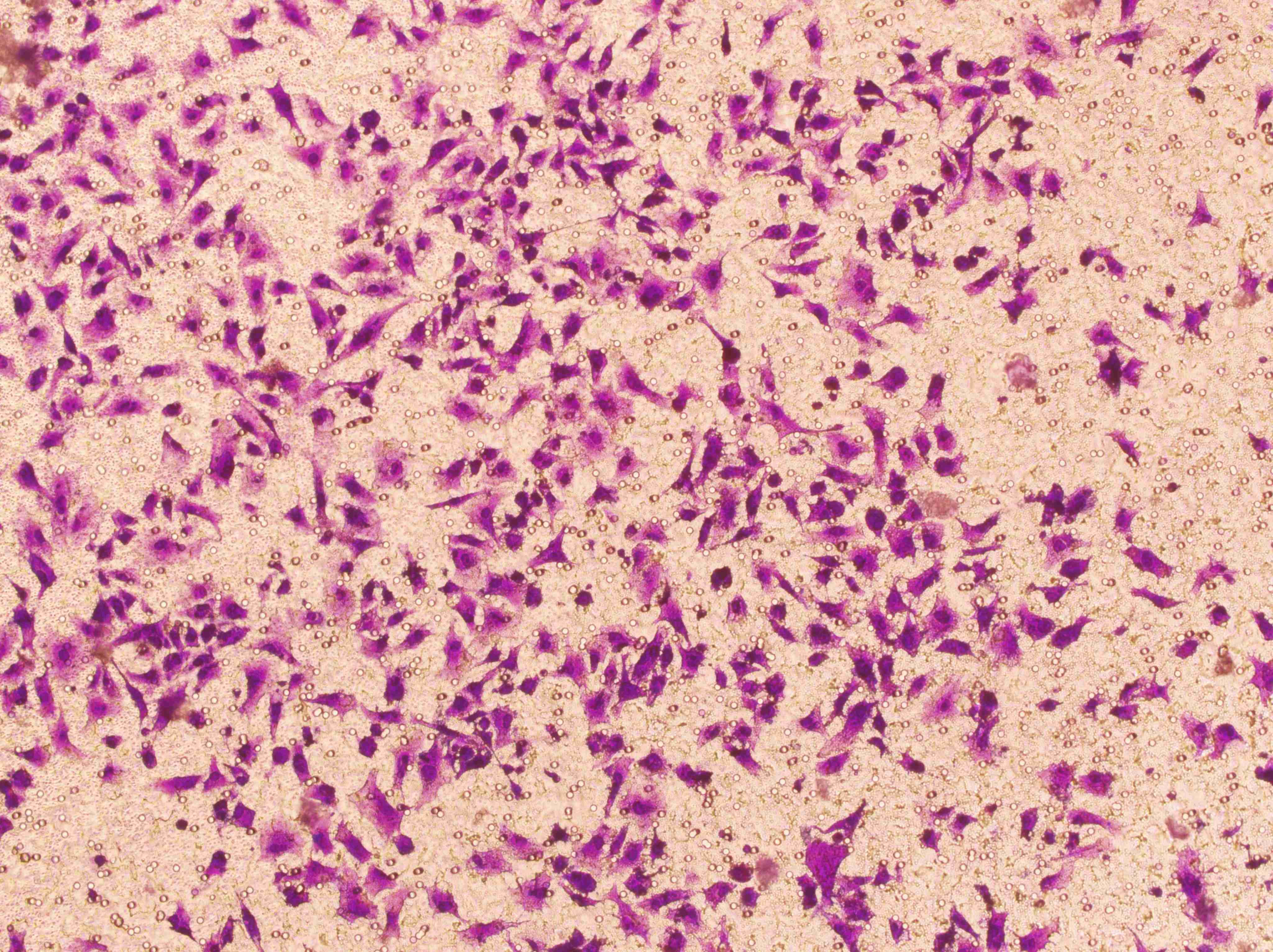

Supplement: Supplementary file 2 [file DataSheet1.zip › Original data/Fig 5G_A549+PBMC_Invasion.jpg]

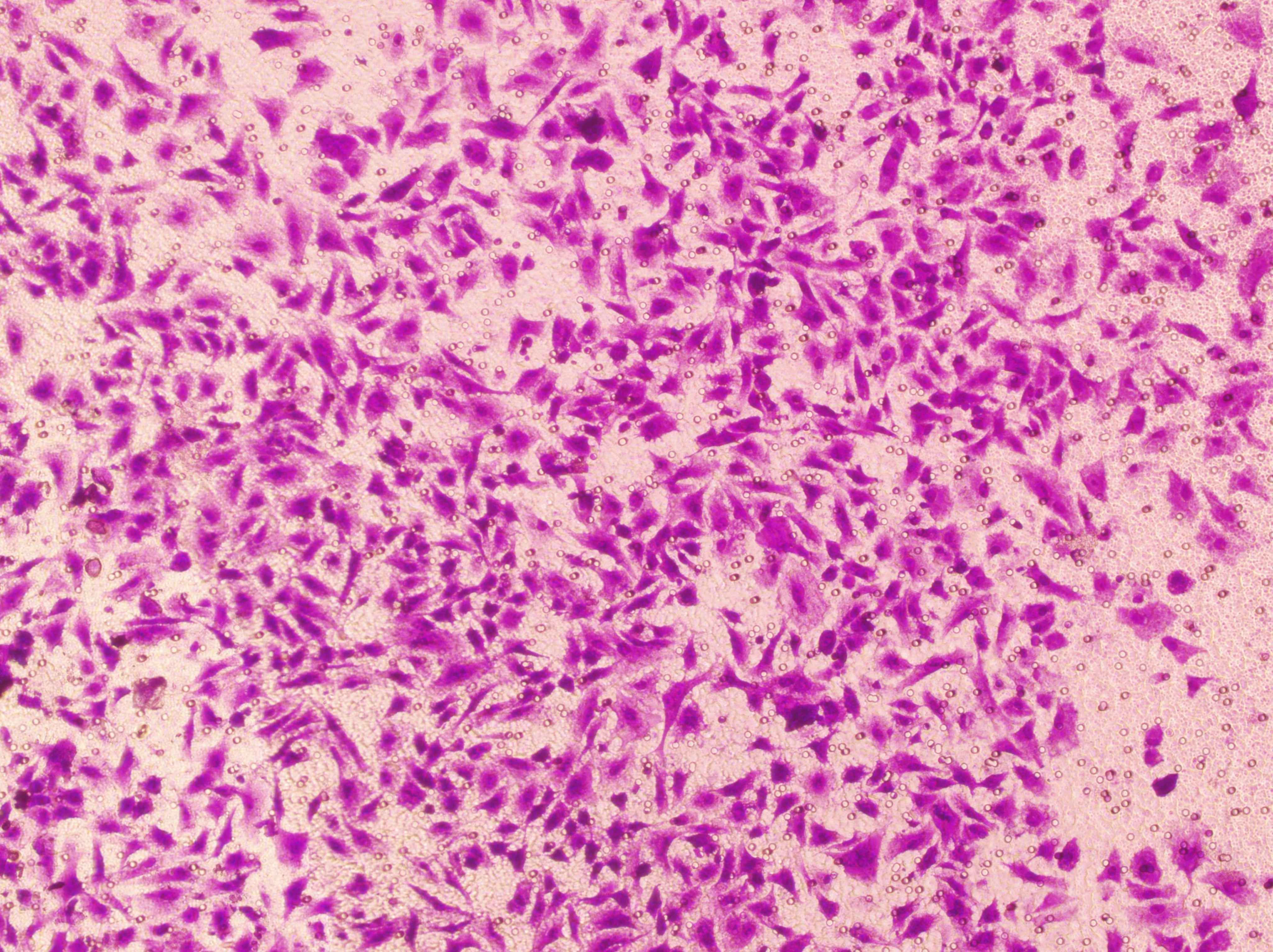

Supplement: Supplementary file 2 [file DataSheet1.zip › Original data/Fig 5G_A549+PBMC_Migration.jpg]

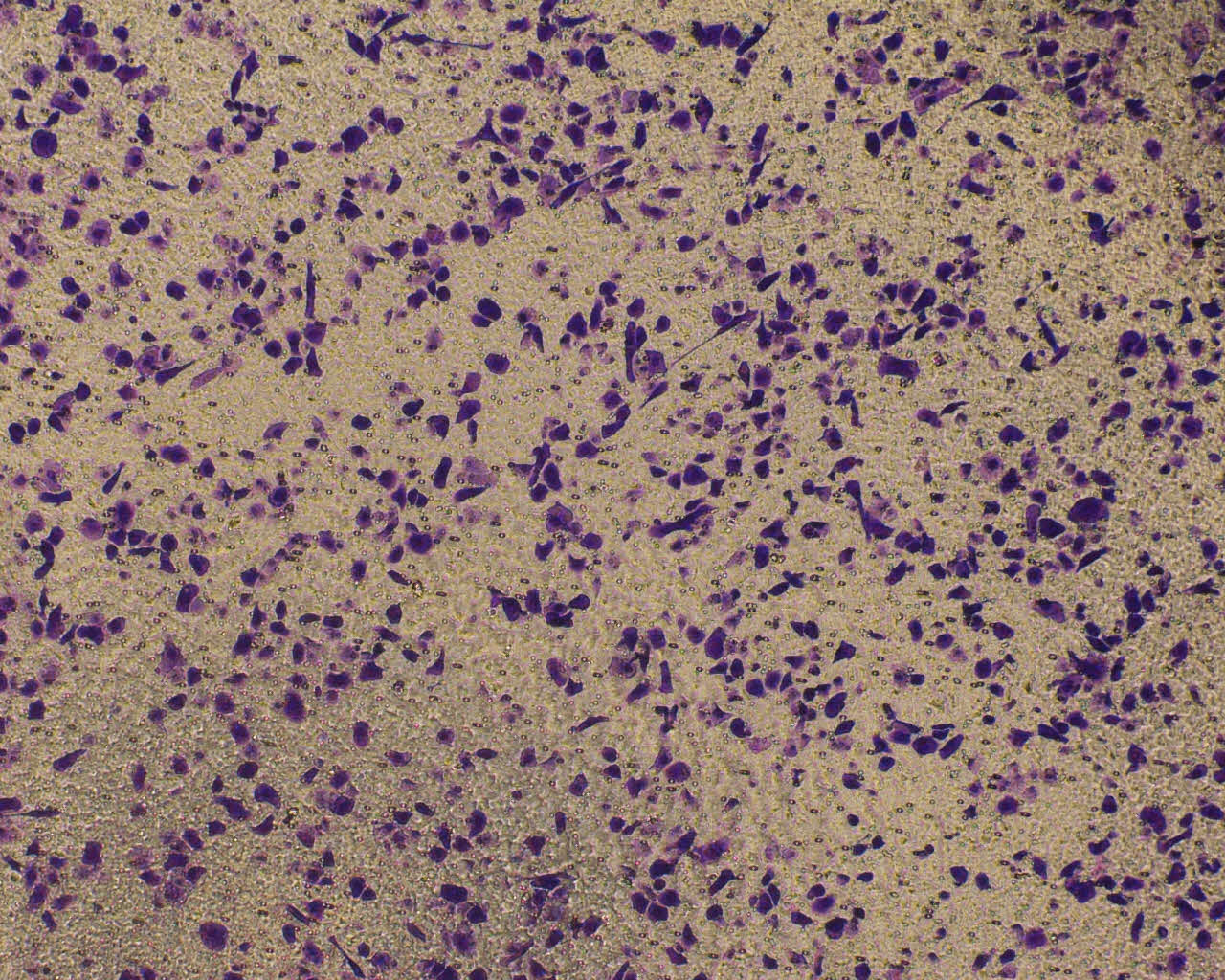

Supplement: Supplementary file 2 [file DataSheet1.zip › Original data/PC9(Poly)+PBMC(Sintilimab)_Migration.jpg]

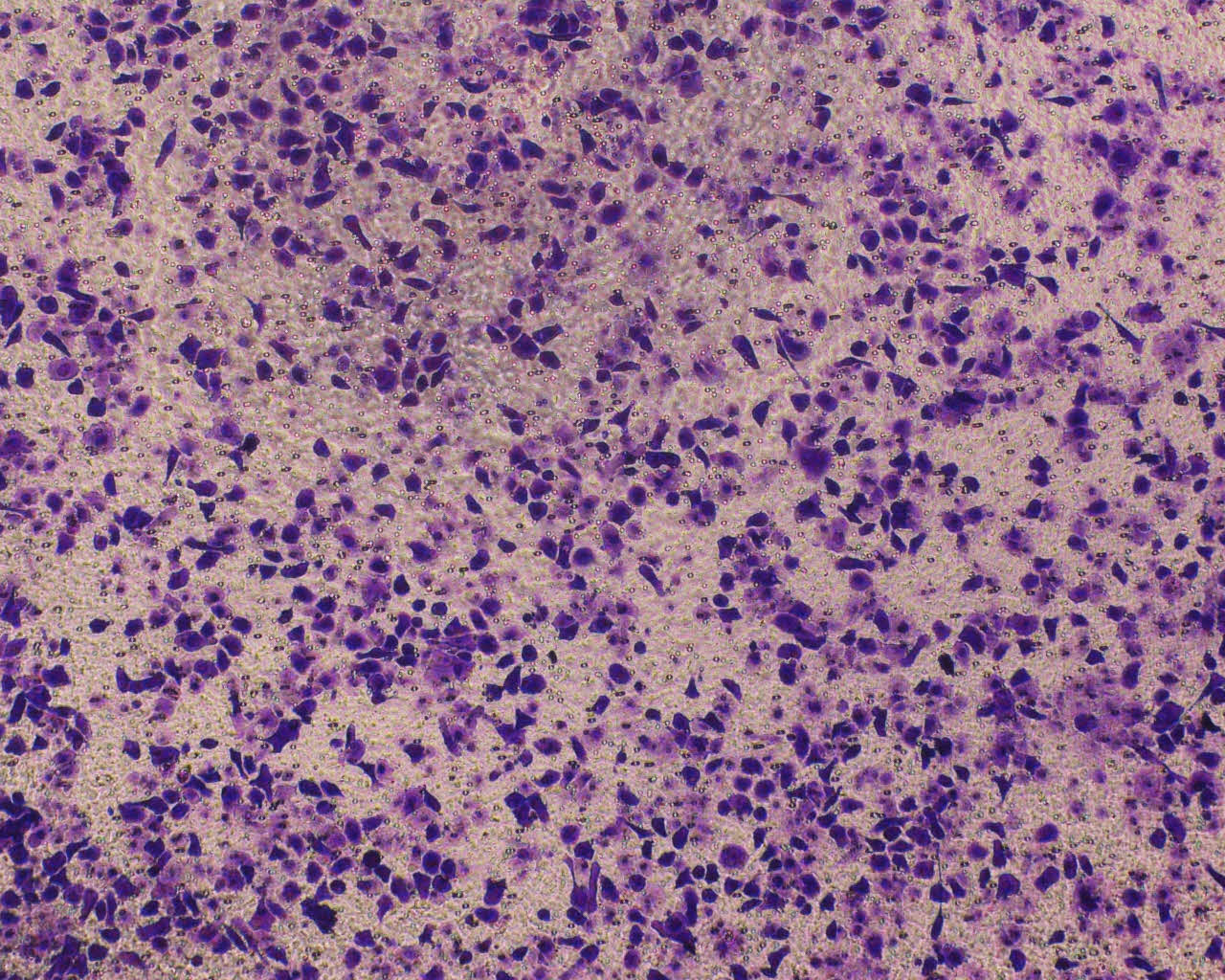

Supplement: Supplementary file 2 [file DataSheet1.zip › Original data/PC9(Poly)+PBMC_Migration.jpg]

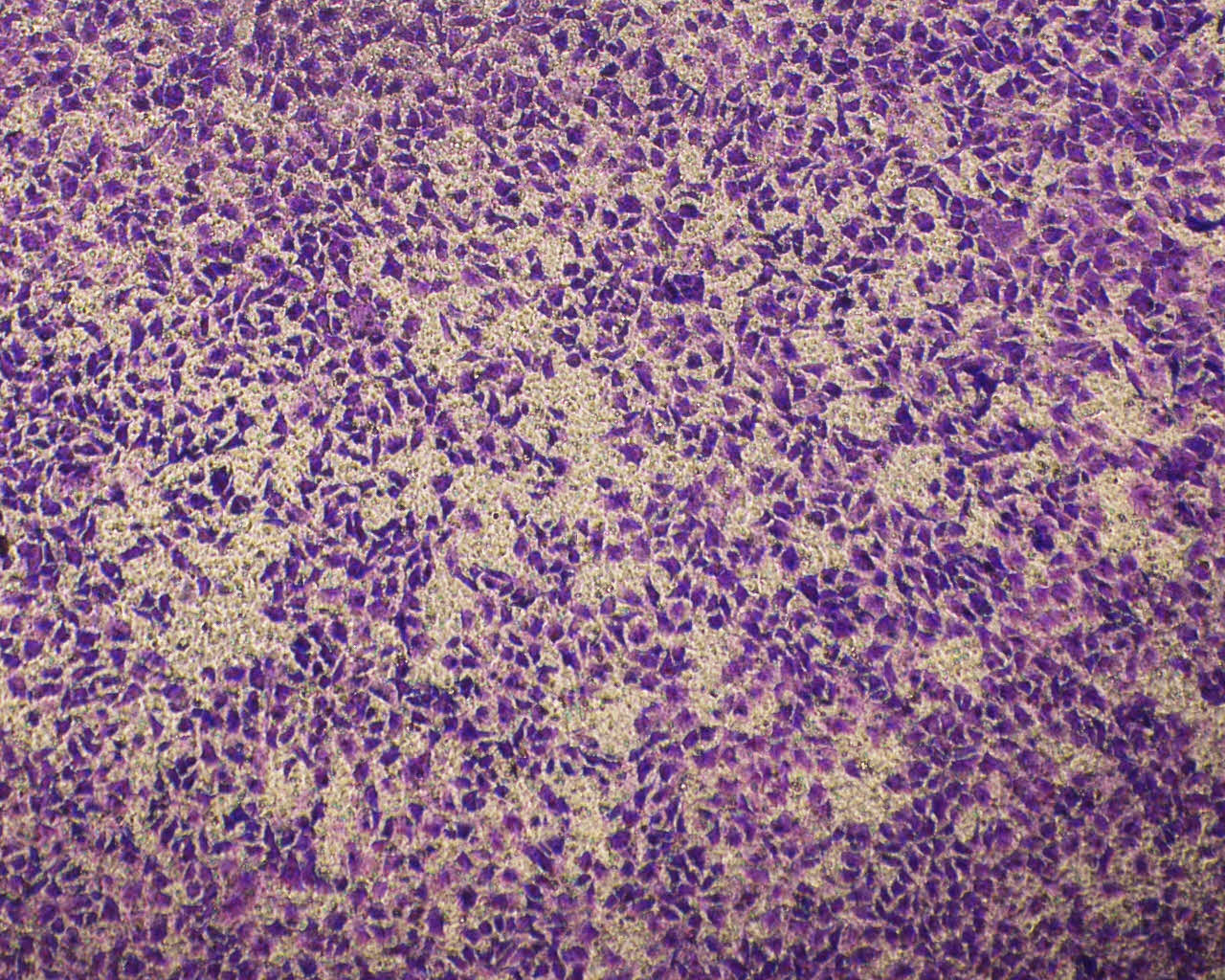

Supplement: Supplementary file 2 [file DataSheet1.zip › Original data/PC9+PBMC(Sintilimab)_Migration.jpg]

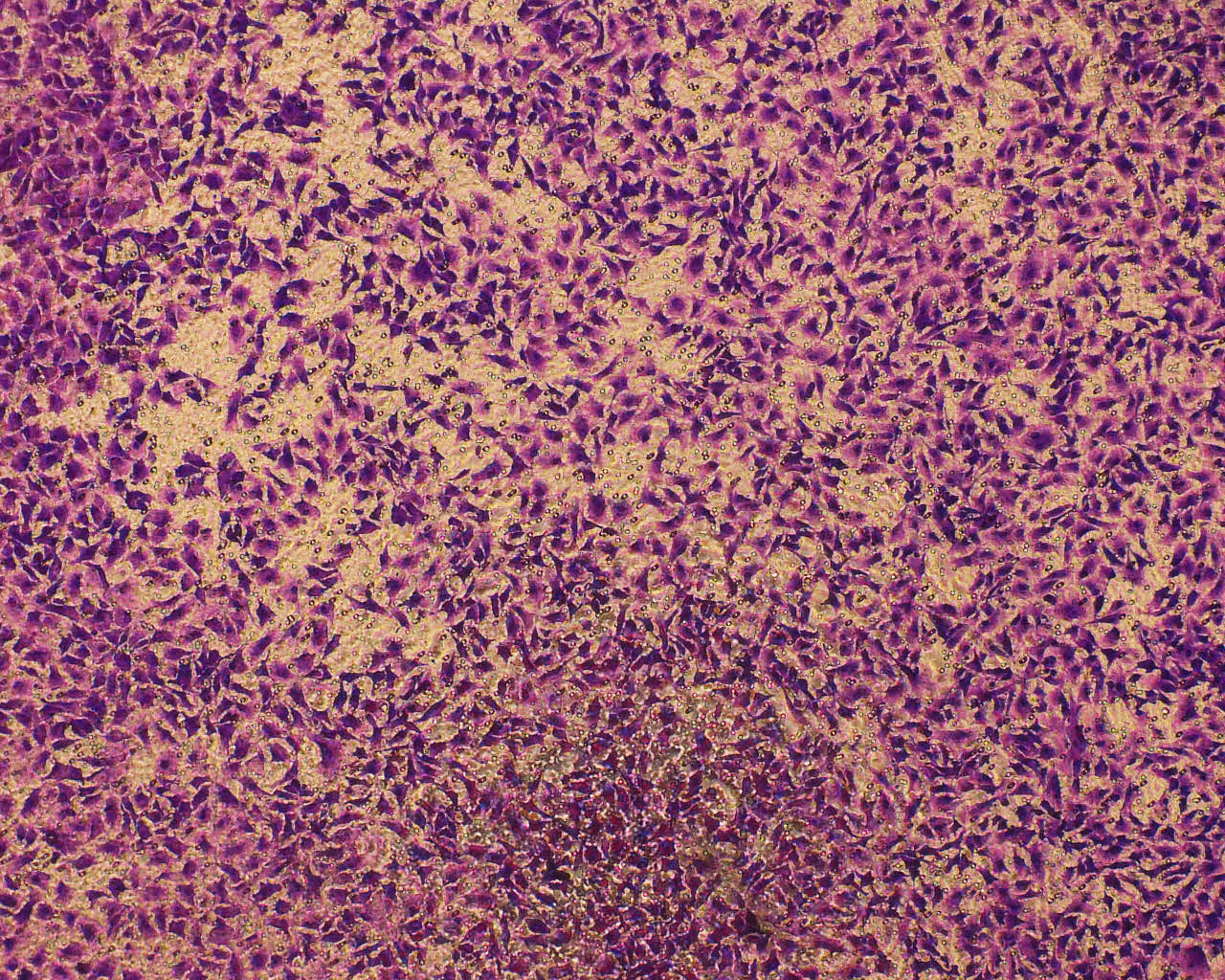

Supplement: Supplementary file 2 [file DataSheet1.zip › Original data/PC9+PBMC_Migration.jpg]
